# Early life stages of stress-tolerant corals (*Favites* spp.) exhibit tolerance to acute thermal stress

**DOI:** 10.1242/bio.062748

**Published:** 2026-10-20

**Authors:** Jeffry A. Jeve, Jacqueline L. Padilla-Gamiño, Maria Vanessa Baria-Rodriguez

**Affiliations:** ^1^ Marine Science Institute, College of Science, University of the Philippines Diliman, 1101 Quezon City, Philippines; ^2^ Bolinao Marine Laboratory, Marine Science Institute, University of the Philippines, 2406 Bolinao, Pangasinan; ^3^ School of Aquatic and Fishery Sciences, University of Washington, Seattle, WA 98195, USA

**Keywords:** Scleractinian coral, Embryo, Larvae, Recruit, High temperature

## Abstract

The Bolinao-Anda Reef Complex (BARC) in northwestern Philippines is one of the most heavily impacted reef systems in the Philippines, experiencing recurring marine heat waves and declining water quality due to coastal development and mariculture. As ocean warming and declining water quality continue, stress-tolerant coral species are expected to become increasingly dominant. Understanding how these corals respond to elevated temperatures in the early stages is critical for predicting future reef trajectories. In this study, we examined survival and development of embryos, settlement and survival of larvae, and survival of recruits of the stress-tolerant corals *Favites abdita* and *Favites colemani* collected from BARC under control (28°C) and elevated (33-35°C) temperatures. In both species, elevated temperature did not significantly affect embryo survival and development, larval survival and settlement, or recruit survival. Results show that early stages of *Favites* development can tolerate temperatures ranging from 28 to 35°C. These findings support the classification of *Favites* spp. as stress-tolerant corals capable of persisting in BARC despite repeated thermal anomalies. While early life stages exhibited short-term thermal tolerance, further research is needed to determine whether thermally exposed recruits experience long-term physiological or ecological trade-offs.

## Teaser

**Summary:** Embryos, larvae, and recruits of the stress-tolerant corals *Favites abdita* and *Favites colemani* can tolerate acute thermal stress (up to 35°C).

## INTRODUCTION

Global coral bleaching events have increasingly devastated reef systems worldwide, causing substantial declines in coral cover, structural complexity, and the ecosystem services that reefs provide (Woodhead et al., 2019; Eddy et al., 2021; Zeng et al., 2025). The frequency and severity of these events have intensified over recent decades due to rising sea surface temperatures (SST), with mass bleaching now occurring at intervals too short for full reef recovery (Hughes et al., 2018; IPCC, 2023). Global assessments estimate that approximately 14% of coral cover was lost between 2009 and 2018, with heat stress as a major contributing driver (Souter et al., 2021), highlighting the cumulative impacts of recurrent disturbances. Beyond reducing coral abundance, repeated bleaching has altered coral community composition, driving declines in fast-growing branching taxa and a relative increase in slower-growing, stress-tolerant massive corals (Marshall and Baird, 2000; Loya et al., 2001; Darling et al., 2012; Kubicek et al., 2019; Pisapia et al., 2019; Chan et al., 2024). Consequently, reef assemblages are increasingly shaped by functional traits associated with stress tolerance, growth, and recovery potential rather than rapid growth or colonization ability (Hughes et al., 2018; McWilliam et al., 2020). These changes have important implications for reef resilience, as repeated bleaching events affect adult coral survival and community structure, reduce reproductive output, and compromise early-life-stage success, ultimately constraining recruitment and long-term population persistence (Ward et al., 2000; Baird and Marshall, 2002). As SSTs are projected to continue rising under future climate scenarios (IPCC, 2023), more frequent bleaching events threaten coral survival, biodiversity, ecological function, and the recovery capacity of reef ecosystems.

Early life-history stages are critical demographic bottlenecks in corals because they regulate recruitment through processes such as larval production, dispersal, settlement, and early-post-settlement survival (Richmond and Hunter, 1990; Ritson-Williams et al., 2009; Doropoulos et al., 2017). These stages are characterized by high mortality and strong environmental sensitivity, making them key determinants of population persistence and recovery trajectories (Cowen and Sponaugle, 2009; Speare et al., 2025). Understanding coral responses during these stages is therefore essential for explaining how community shifts emerge and persist following disturbance, as successful recruitment ultimately governs population replenishment and reef recovery (Mumby and Steneck, 2008; Ritson-Williams et al., 2009; Holbrook et al., 2018). In contemporary reef systems, recruitment success is increasingly constrained under climate-driven stress, with marine heat waves (MHWs) shown to significantly reduce larval supply and settlement success (Hughes et al., 2019; Figuiredo et al., 2022). As a result, both natural recruitment processes and restoration approaches, such as larval enhancement, are receiving increasing attention as strategies to support reef recovery under a warming climate (Heyward et al., 2002; dela Cruz and Harrison, 2017, 2020).

Despite the ecological importance of corals, relatively little is known about how their early life stages respond to progressive ocean warming, particularly under increasingly frequent and prolonged elevated temperature conditions projected for the coming decades (IPCC, 2023). Existing studies show considerable variation among species and developmental stages, with thermal stress affecting embryonic, larval, and recruit survival and larval settlement success in different ways (Table 1). Moreover, information on the early-life-stage thermal tolerance of stress-tolerant coral species remains scarce. It is unclear whether offspring of thermally resilient adult colonies consistently exhibit similar tolerance under elevated temperatures (Quigley et al., 2020; McRae et al., 2021). It is also uncertain whether experimental temperature regimes used in laboratory experiments adequately reflect ecologically realistic warming conditions during MHWs (Oliver et al., 2018; Smale et al., 2019). Nevertheless, controlled laboratory experiments remain essential for isolating temperature effects, identifying thermal thresholds, and quantifying developmental sensitivity across early life stages, insights that are difficult to obtain under variable field conditions. Addressing these gaps is essential for improving predictions of recruitment dynamics and long-term coral population persistence under ongoing ocean warming (Hughes et al., 2017; IPCC, 2023).

**Table 1.** Summary of experimental studies examining the responses of different hard coral early life stages (embryos, larvae, and recruits) to thermal stress under controlled conditions

Reference	Species	Suborder	Family	Source of donor colony	Early life stage (age)	Temperature treatments (°C)	Exposure duration	Effects
**Embryos**								
Negri et al. (2007)	Acropora millepora ^C,*^	Refertina	Acroporidae	Australia	Gametes to embryos	28.3 (ambient), 30.4, 31.8, 32.8, and 34.1 (range±0.5)	2-4 h	− (Fertilization rate and embryonic development)
	Favites abdita ^ST,*^	Vacatina	Merulinidae					= (Fertilization and embryonic development)
	Mycedium elephantotus ^G,*^	Vacatina						= (Fertilization and embryonic development)
	Favites chinensis ^G,*^	Vacatina		Japan		25.5, 26.4, 27.0 (ambient), 27.8, 28.9, 30.2, 31.6 (range±0.5)		= (Fertilization and embryonic development)
Bassim et al. (2002)	Pseudodiploria strigosa ^ST,*^ formerly *Diploria*	Vacatina	Faviidae	Mexico	Embryos	30 (ambient), 31, 32	24 h	− (Embryonic development)
Randall and Szmant (2009a)	Acropora palmata ^C,*^	Refertina	Acroporidae	Mexico	Embryos (2- to 16-cell stage)	28 (ambient), 30, 31.5	7 days	+ (Embryonic development at 30°C and 31.5°C)−(high embryonic aberrations at 31.5°C)−(embryonic survivorship at 30 and 31.5°C)
Keshavmurthy et al. (2014)	Acropora muricata ^C,*^	Refertina	Acroporidae	Taiwan	Embryos	15, 20, 25, 28 (ambient), and 33	48 h	− (Delayed fertilization at 15°C)+(fertilization rate at 33°C)
	Acropora hyacinthus ^C,*^	Refertina						− (Delayed embryonic development at 20°C) −(Survival at 15 and 33°C)
Woolsey et al. (2015)	Acropora millepora ^C,*^	Refertina	Acroporidae	Australia	Embryos to larvae	Ambient (22, 24, 28)±2 and 4 (five treatments including ambient)	144 h	− (High embryonic aberrations under elevated temperatures)−(life span at elevated temperatures and −4°C)
	Acropora spathulata ^C,*^	Refertina						− (High embryonic aberrations under elevated temperatures)−(life span at elevated temperatures)
	Cyphastrea microphthalma ^G,*^	Vacatina	Merulinidae					− (High embryonic aberrations under elevated temperatures)−(life span at +4°C)
	Goniastrea favulus ^G,*^	Vacatina						− (High embryonic aberrations under elevated temperatures at Lizard Island)=(life span at elevated temperatures)
	Paragoniastrea australensis ^G,*^ formerly *Goniastrea*	Vacatina						− (High embryonic aberrations under elevated temperatures)−(life span at +4°C)
Chua et al. (2013)	Acropora millepora ^C,*^	Refertina	Acroporidae	Australia	Gametes to larvae	27 (ambient) and 29	96 h	− (Fertilization of Nov_22 assay) =(fertilization of Nov_01 and Nov_02 assay)
	Acropora tenuis ^C,*^	Refertina						= (Fertilization)+(rate of development)
This study	Favites abdita ^ST,*^	Vacatina	Merulinidae	Philippines	Embryos (4- to 16-cell stage; ∼4 h post-fertilization)	28 (control) and 33	∼24 h	= (Rate of development)=(survival)
	Favites colemani ^ST,*^	Vacatina						
**Larvae**								
Randall and Szmant (2009b)	Favia fragum ^ST,**^	Vacatina	Faviidae	Curaçao	Larvae	28 (control), 29, and 31	∼6.5 days	− (Larval survival and settlement at 31°C)
Randall and Szmant (2009a)	Acropora palmata ^C,*^	Refertina	Acroporidae	Mexico	Pre-exposed larvae (7 days post-fertilization)	28 (ambient), 30, 31.5	7 days (exposed as embryo)+∼7.5 days (larva to settlement)	− (Larval survivorship and settlement at 30 and 31.5°C)
Heyward and Negri (2010)	Acropora millepora ^C,*^	Refertina	Acroporidae	Australia	Larvae	26, 28 (ambient), 30, and 32 (±0.3)	Up to 6 days	= (Larval survival at 30°C)−(larval survival at 32°C)=(larval settlement competency at 32°C after 4 days)
	Acropora spathulata^C,*^	Refertina						= (Larval survival at 30 and 32°C) |−(larval settlement competency at 32°C after 4 days)
	Lobophyllia recta ^ST, *^ formerly *Symphyllia*	Vacatina	Lobophylliidae					= (Larval survival at 30°C) −(larval survival at 32°C) =(larval settlement competency at 32°C after 4 days)
	Lithophyllon repanda ^G,*^ formerly *Fungia*	Vacatina	Fungiidae	Japan		26, 28 (ambient), and 30 (±0.5)	4 days	+ (Larval settlement competency)
Chua et al. (2013)	Acropora millepora ^C,*^	Refertina	Acroporidae	Australia	Larvae (5-day-old)	27 (ambient) and 29	14 days	− (Larval survivorship)−(larval metamorphosis)
	Acropora tenuis ^C,*^	Refertina						− (Larval survivorship) |+(larval metamorphosis)
Graham et al. (2017)	Acropora tenuis ^C,*^	Refertina	Acroporidae	Australia	Larvae	25.0, 27.0 (ambient), 28.5, 30.0, and 31.5	Up to 54 days	− (Increased mortality rates with increasing temperature)
Weeriyanun et al. (2022)	Acropora tenuis ^C,*^	Refertina	Acroporidae	Australia	Larvae	27.5 (control) and 35.5	48 h	− (Larval settlement in all crosses)
Da-Anoy et al. (2020)	Acropora tenuis ^C,*^	Refertina	Acroporidae	Philippines	Larvae	28.01±0.02 (control), 30.58±0.01, and 32.42±0.02	24 h	− (Larval survival and altered settlement cue preference at the highest temperature)
Baird et al. (2006)	Acropora muricata ^C,*^	Refertina	Acroporidae	Japan	Larvae	28 (ambient), 32, and 36	7 days	= (Larval survival at 32°C)−(larval survival at 36°C)
Baria et al. (2015)	Fungia fungites ^C,*,**^	Vacatina	Fungiidae	Japan	Larvae	28 (ambient), 31, and 34	8 days	= (Larval survival)
	Lithophyllon repanda ^G,*^	Vacatina						− (Larval survival at 34°C)
Schnitzler et al. (2012)	Lobactis scutaria ^G,*^ formerly *Fungia*	Vacatina	Fungiidae	USA (Hawai'i)	Larvae	27 (ambient), 29, and 31	72 h	− (Larval survivorship under elevated temperatures)−(larval survivorship with symbionts at 31°C)−(larval algal symbiont infection at 31°C)
Huffmyer et al. (2024)	Montipora capitata ^C,*^	Refertina	Acroporidae	USA (Hawai'i)	Larvae	27 (control) and 30	3 days	= (Larval survival and settlement)−(reduced larval respiration)
Cumbo et al. (2013)	Pocillopora damicornis ^W,*,**^	Vacatina	Pocilloporidae	Taiwan	Larvae	24 (Ambient) and 30.49	1-5 days	= (Protein content and *Symbiodinium* density)+(respiration)−(larval survival)
Edmunds et al. (2001)	Porites astreoides ^W,**^	Refertina	Poritidae	USA (Florida Keys)	Larvae	∼26, 28 (ambient), and 33	24 h	− (Larval survival at 32°C)+(larval metamorphosis at 32°C)=(larval motility)−(gross photosynthesis at ∼26 and 32°C)
Olsen et al. (2013)	Porites astreoides ^W,**^	Refertina	Poritidae	USA (Florida Keys)	Larvae (1-day-old)	27.3 (control) and 30.8	4-48 h	= (Larval survival and settlement)−(reduced larval respiration rates)
Putnam et al. (2008)	Stylophora pistillata ^W,**^	Vacatina	Pocilloporidae	Taiwan	Larvae	22.6, 25.4 (ambient), and 28.6	∼12 h	= Larval photosynthetic efficiency (Fv/Fm)+(increased settlement on crustose coralline algae)
This study	Favites abdita ^ST,*^	Vacatina	Merulinidae	Philippines	Larvae (1-day-old)	28 (control) and 33	∼24 h	= (Larval survival)=(larval settlement)
	Favites colemani ^ST,*^	Vacatina						
**Recruits**								
Hazraty-Kari et al. (2023)	Acropora tenuis ^C,*^	Refertina	Acroporidae	Japan	Thermally conditioned larvae to recruits (up to 42 days)	∼26 (control) and ∼31	up to 28 days	= (Recruit survival) =(Recruits from thermal stress-conditioned larvae)
Alexander et al. (2022)	Montipora capitata ^C,*^	Refertina	Acroporidae	USA (Hawai'i)	Thermally conditioned larvae to recruits (1-month old)	24.5±1.0, 27.2±0.3 (ambient), and 28.9±0.7	4 days (exposed as larvae) and 47 days (recruits)	− (Larval survival outside ambient temperature)+(larval settlement under warm temperature)−(thermally conditioned recruit survivorship)
Nozawa and Harrison (2007)	Favites chinensis ^ST,*^	Vacatina	Merulinidae	Japan	Larvae to recruits (up to 5 days)	27 (ambient) and 31 or 34	1 h	+ (Larval settlement)=(post-settlement mortality)
	Acropora solitaryensis ^C,*^	Refertina	Acroporidae	Australia		20, 23 (ambient), 26 and 29	5 days	+ (Larval settlement)−(high post-settlement mortality at 29°C)
Chui and Ang (2017)	Platygyra acuta ^ST,*^	Vacatina	Merulinidae	Hong Kong	Larvae and recruits	24, 27 (ambient), 30, and 32	Up to 56 days	= (Post-settlement survival under elevated temperatures)+(larval settlement success under elevated temperature)
Lamb et al. (2024)	Acropora kenti ^C,*^×Acropora loripes ^C,*^	Refertina	Acroporidae	Australia	Recruits (5-month-old)	27.8 (control) and 30.6	5 weeks	= (Recruit survivorship, size, color and photochemical efficiency of photosystem II)
	Acropora kenti ^C,*^	Refertina						
	Acropora loripes ^C,*^	Refertina						
Huffmyer et al. (2021)	Pocillopora acuta ^W,* and **^	Vacatina	Pocilloporidae	USA (Hawai'i)	Recruits (15-day-old)	27.3±0.5 (ambient) and 29.8±1.3	32 days	− (Recruit survival)
This study	Favites abdita ^ST,*^	Vacatina	Merulinidae	Philippines	Recruits (1-day-old)	28 (control) and 35	∼24 h	= (Recruit survival)
	Favites colemani ^ST,*^	Vacatina						

Note: (=) no difference, (+) positive effect, (−) negative effect from the control or ambient.

Suborder based on Okubo (2016). Life history strategies based on Darling et al. (2012): ^C^Competitive, ^ST^Stress-tolerant, ^W^Weedy, ^G^Generalist. Sexual reproduction strategies: *Broadcast spawner and **Brooder; ambient/control.

In the Philippines, the Bolinao-Anda Reef Complex (BARC) represents one of the country's best-studied reef systems due to its proximity to the Bolinao Marine Laboratory of the Marine Science Institute, University of the Philippines Diliman (BML MSI-UPD). However, only a few studies focused on coral early life stages, and all were conducted at BARC. These include manipulation of sperm concentration and fertilization time in *Acropora millepora*, *Acropora tenuis*, and *Favites colemani* (dela Cruz and Harrison, 2019); herbivore exclusion and depth effects in *A. tenuis* recruits (Baria et al., 2010); larval density effects in *A. tenuis* (Cameron and Harrison, 2020); maternal tissue mortality effect on offspring performance in *A. tenuis* (Dumato et al., 2026); temperature effects on larval settlement cues in *A. tenuis* (Da-Anoy et al., 2020); recruit density effects in *Acropora verweyi* (Ligson et al., 2022); and fish farm effluent impacts in *Pocillopora acuta* (Quimpo et al., 2020a). Although these studies have improved our understanding of coral early life stages in BARC, important knowledge gaps remain regarding the effects of elevated temperature on Philippine corals. This gap reflects a broader global pattern, where experimental studies examining thermal responses during the early life stages of stress-tolerant coral species remain scarce, particularly for *Favites abdita* and *F. colemani*. Although evidence shows that adult *F. colemani* can tolerate warming (Tañedo et al., 2021), responses of the early life stages of both *Favites* species to elevated temperatures remain largely unexplored.

Moreover, BARC is a recognized MHW hotspot (Edullantes et al., 2023) and has experienced bleaching events of varying intensity, including widespread bleaching after the 1998 global event (Arceo et al., 2001) and lower bleaching prevalence during the 2016 thermal stress event (Quimpo et al., 2020b). More recently, thermal anomalies associated with the 2023-2024 El Niño contributed to additional bleaching and declines in hard coral cover (Francisco et al., 2025, MHW Project, unpublished). These reefs also experience chronic local stressors, including nutrient enrichment and impacts from mariculture (San Diego-McGlone et al., 2008; Ferrera et al., 2016; Isah et al., 2022). Despite these pressures, stress-tolerant taxa, including massive corals, persist at BARC (MHW Project, unpublished; personal communication). However, repeated bleaching events may still increase the risk of mortality even in these resilient groups (Grottoli et al., 2014; Slattery et al., 2019). The persistence of adult colonies does not necessarily indicate comparable thermal resilience in their offspring, as parental thermal conditioning can have neutral, positive, or even adverse effects on offspring performance under subsequent thermal stress (McRae et al., 2021; Lamb et al., 2026).

*F.* *abdita* (Ellis and Solander, 1786) and *F. colemani* (Veron, 2000) are common reef-building corals in BARC. Both species are currently listed as ‘Least Concern’ on the International Union for Conservation of Nature (2025, https://www.iucnredlist.org/) Red List with a decreasing population trend (Huang et al., 2024; Shlesinger et al., 2024). They belong to the family Merulinidae, exhibit sub-massive to massive morphologies (Fig. 1, WoRMS Editorial Board, 2026, https://www.marinespecies.org/aphia.php?p=taxdetails&id=1363), are hermaphroditic broadcast spawners and are widely distributed in the Indo-Pacific (depths of 0-47 m), including the Philippines (Ross and Hodgson, 1981; Veron, 2000; Maboloc et al., 2015; Ocean Biodiversity Information System, 2025, https://obis.org). *F. abdita* can reach up to 1 m in diameter (Madin et al., 2016, https://www.coraltraits.org/species/670), whereas *F. colemani* may reach up to 40 cm (Madin et al., 2016, https://www.coraltraits.org/species/976). Although colonies of *Favites* were negatively affected by the 2024 bleaching event, they remain present in BARC (MHW Project, unpublished; personal communication). These species are particularly suitable for experimental studies due to their predictable spawning schedules and well-established larval rearing protocols (Maboloc et al., 2015; Bonilla et al., 2021, 2023; Guest et al., 2023). Both species have overlapping spawning windows between 20:30 and 22:30 h, allowing simultaneous comparative experimentation (Maboloc et al., 2015).

**Fig. 1.**
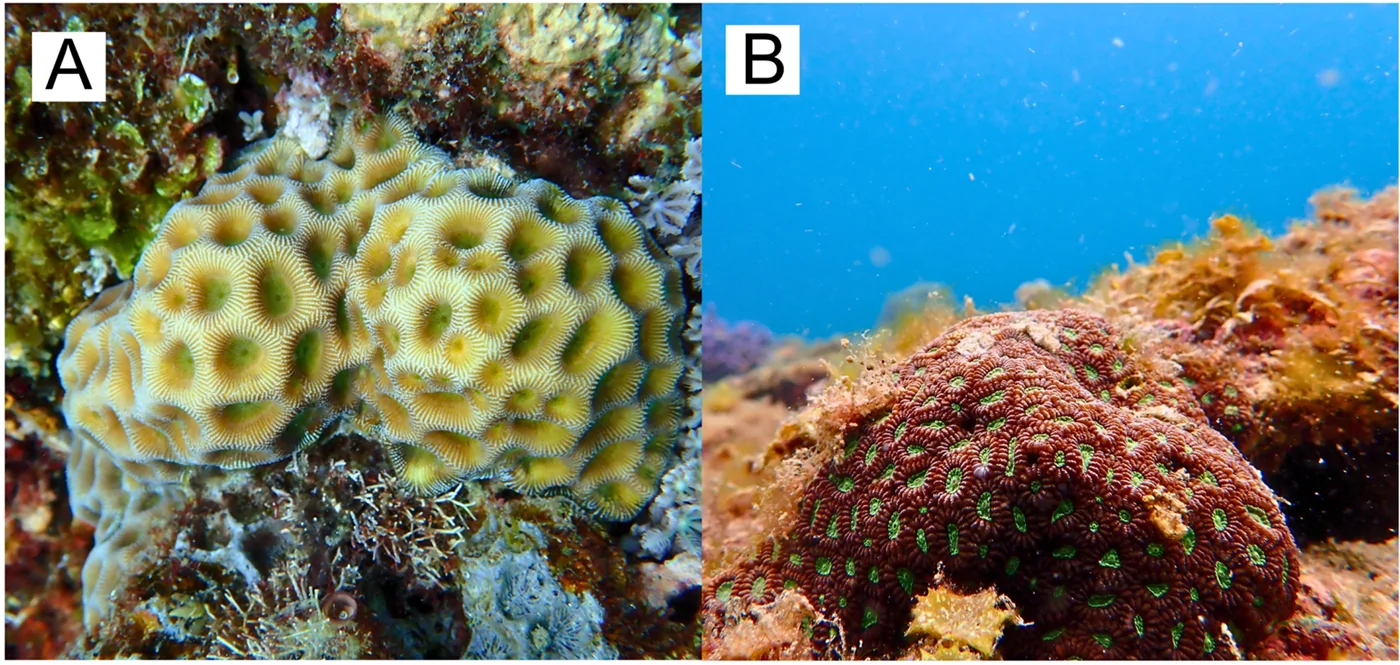
**Stress-tolerant hard coral species.** (A) *Favites abdita* and (B) *F. colemani.* Images taken using an OM System Tough TG-7.

This study investigated how elevated temperature affects the early life stages of *F. abdita* and *F. colemani* under controlled laboratory conditions. Specifically, we aimed to (1) evaluate the survival and development of embryos, (2) assess larval survival and settlement, and (3) determine recruit survival under increased temperature. We hypothesized that increased temperature would negatively impact embryo development and the larval and recruit stages in both species, particularly when temperatures are 5-7°C above ambient during spawning. With reefs experiencing recurring thermal stress and declining water quality, understanding the performance of *F. abdita* and *F. colemani* under elevated temperatures can provide insight into whether stress-tolerant taxa will continue to contribute to natural reef recovery under future ocean warming scenarios.

## RESULTS

*F. colemani* crosses yielded an estimated 206,556 eggs, with 65.61% being successfully fertilized, whereas *F. abdita* crosses produced 35,378 eggs with a fertilization rate of 63.32%. Responses of early life stages (embryos, larvae, and recruits) to short-term elevated temperatures (33 and 35°C) were assessed in independent, stage-specific experiments. Embryonic development and survival, larval survival and settlement, and recruit survival were measured under control and elevated-temperature conditions.

### Embryo survival and development

Both *F. abdita* and *F. colemani* exhibited high embryo survival (>90%) at an increased temperature (Fig. 2A). No significant differences in survival were observed between control and increased temperature for either species (*F. abdita*: *r*=0.36, *P*=0.50; *F. colemani*: *r*=0.19, *P*=0.81, Wilcoxon rank-sum test; Tables S3 and S5). Embryo-to-planula development was not affected by temperature, with both species exhibiting 100% development to actively swimming planula at ∼20 h in both control and elevated-temperature treatments, even before reaching the target elevated temperature (Fig. 3C).

**Fig. 2.**
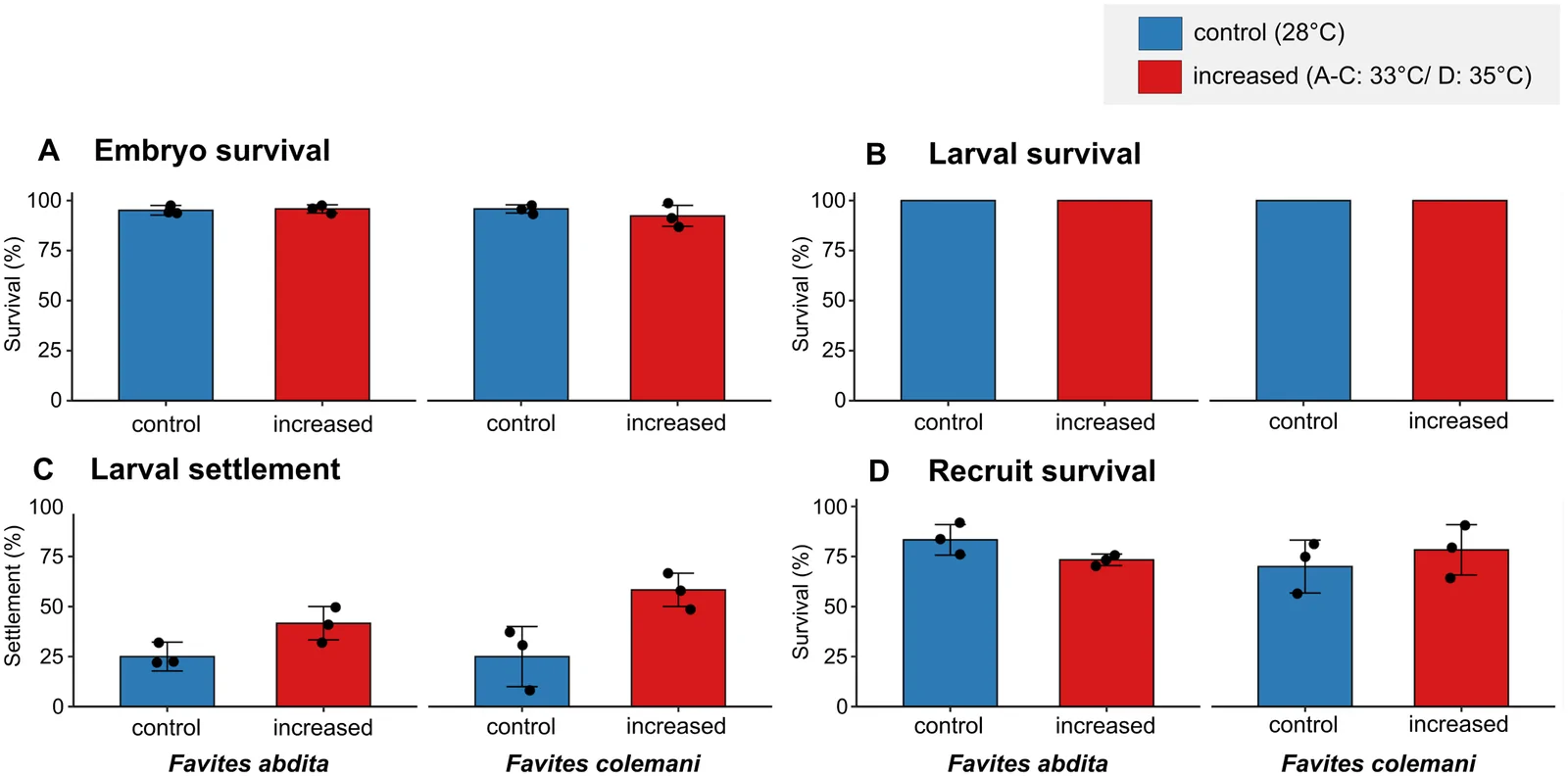
**Effects of elevated temperature on early-life-history performance of *F. abdita* and *F colemani.*** (A) Embryo survival, (B) larval survival, (C) larval settlement, and (D) recruit survival under control (blue bars; 28°C) and elevated (red bars; 33 or 35°C) temperature. Bars show mean±s.d. (*n*=3 per temperature treatment). No significant differences (*P*>0.05) were detected between the two temperature treatments in either species for all measured parameters.

**Fig. 3.**
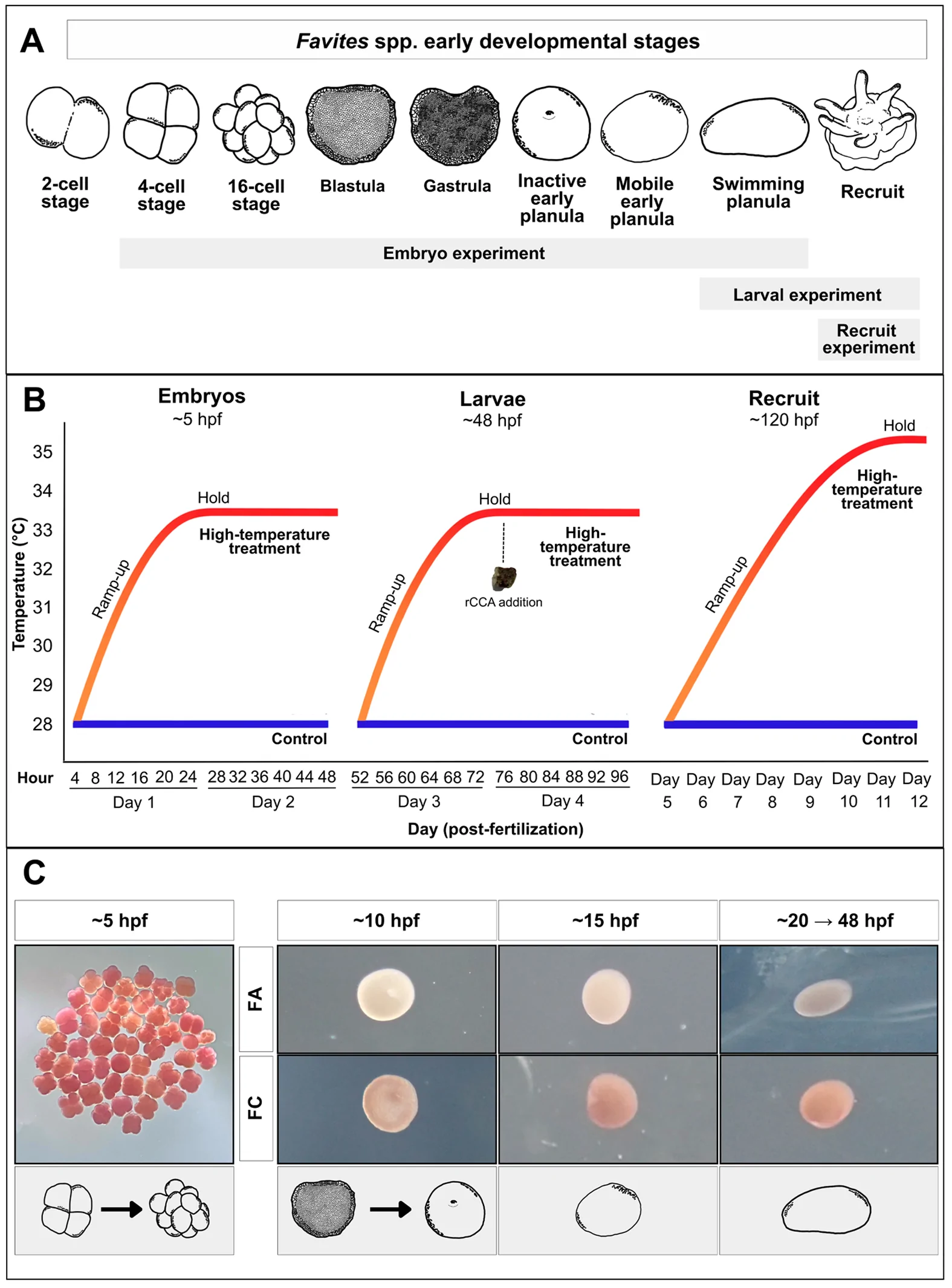
**Experimental overview and early developmental stages of *F. abdita* and *F. colemani* during thermal exposure experiments.** (A) Early developmental stages used in independent thermal stress experiments, highlighting stage-specific exposure windows for embryos, larvae, and recruits. (B) Temperature profiles showing control (28°C) and ramped and held target temperatures (33 and 35°C). rCCA; rubble with crustose coralline algae; hpf, hours post-fertilization. (C) Representative embryonic developmental stages of *F. abdita* (FA) and *F. colemani* (FC) under control and elevated temperature conditions (28 to 33°C ramp-up exposure), shown for qualitative illustration. Images were acquired using an iPhone 13 Pro through a stereomicroscope (first panel) and an OM System Tough TG-7 (second to fourth panels).

### Larval survival and settlement

Both *F. abdita* and *F. colemani* exhibited 100% survival under both control and elevated-temperature treatments (Fig. 2B).

Settlement tended to be higher under elevated temperature conditions (33°C) for both species compared to the control treatment (28°C) (Fig. 2C). In *F. abdita*, settlement was 25.00±15.02% in the control treatment and 58.33±8.33% in the elevated temperature (*r=*0.80, *P*=0.10, Wilcoxon rank-sum test; Tables S4 and S5). Similarly, *F. colemani* showed settlement values of 25.0±7.22% in the control treatment and 41.67±8.33% in the elevated temperature (*r=*0.73, *P*=0.11, Wilcoxon rank-sum test; Tables S4 and S5). Although settlement tended to be higher under elevated temperature, the differences were not statistically significant for either species.

### Recruit survival

In *F. abdita*, coral recruits exposed to elevated temperature (35°C) exhibited slightly higher survival (78.33±12.58%) compared to the control (70.00±13.23%; *r*=0.36, *P*=0.50, Wilcoxon rank-sum test; Tables S3 and S5). In contrast, *F. colemani* showed lower survival in the elevated temperature (73.33±2.89%) than in the control (83.33±7.64%; *r=*0.66, *P*=0.16, Wilcoxon rank-sum test; Tables S3 and S5). However, these differences were not statistically significant (Fig. 2D).

## DISCUSSION

Embryos, larvae, and early recruits of *F. abdita* and *F. colemani* tolerated short-term elevated temperatures of 33°C (embryo and larval stages) and 35°C (recruit stage). These results suggest that the early developmental stages can tolerate acute exposure to temperatures that can induce stress in adult corals. While the environmental history of the parent colonies may have contributed to this response, the extent of such effects remains uncertain and likely interacts with species-specific physiological traits and intrinsic thermal tolerance mechanisms (Putnam and Gates, 2015; Wong et al., 2021). Regardless of the underlying mechanisms, the observed thermal tolerance of early life stages may have important implications for the persistence and recruitment of corals in increasingly disturbed reef systems.

In the present study, *Favites* spp. exhibited thermal tolerance across early life stages. However, this tolerance cannot be attributed solely to the recurrent thermal anomalies experienced by the source colonies from BARC (Edullantes et al., 2023; Francisco et al., 2025). Although parental exposure to thermal stress can influence offspring performance, such effects are not consistently observed and can be neutral or highly variable (McRae et al., 2021; Lamb et al., 2026). Thus, the observed thermal tolerance may instead reflect parental history and contribution. Although we initially collected from both Caniogan and Lucero, the final experimental pool was limited to a few individuals that spawned synchronously and produced sufficient propagules, so most colonies came from Lucero. Because Lucero is close to localized mariculture activities, these parental populations may have localized environmental conditioning or selection pressures that influence offspring performance. Although our study provides a valuable baseline for these populations, future work spanning broader geographic distributions is required to determine if colonies from less-exposed sites exhibit different thermal tolerance thresholds, as colonies sourced from warmer habitats have been shown to yield offspring with higher survival rates under heat stress (Nobre et al., 2026).

Furthermore, because the gametes were bulk pooled immediately after spawning regardless of individual colony fecundity, the final larval cohort likely reflects a disproportionate contribution from more fecund colonies (e.g. *F. colemani* colony 002 and *F. abdita* colony 010; Table S1). While this approach generated sufficient reproductive material to conduct the experiments and provided a realistic snapshot of the wild population on a given spawning night, it prevented us from distinguishing individual maternal effects or quantifying the specific genetic contributions of each parental colony. Because identifying heat-tolerant individuals and accounting for maternal effects are critical to understanding offspring responses to thermal stress (Lenz et al., 2025), future selective breeding efforts using controlled crosses among individual colonies will be essential to disentangle parental contributions to offspring thermal tolerance within the BARC framework.

Fertilization rates should also be interpreted with caution, as they were quantified only 1 h after sperm washing to allow immediate selection of fertilized eggs for the embryo thermal experiment. Fertilization may have continued beyond this period, and reassessment at a later time may have yielded higher estimates. Faviid gametes from both Okinawa and Great Barrier Reef populations also exhibited the thermal tolerance of *Favites* embryos, larvae, and recruits in BARC (Negri et al., 2007). None were affected by seawater temperatures approximately 5-7°C above typical spawning temperatures. This suggests that faviids may exhibit relatively high thermal thresholds during early development, potentially reflecting a stress-tolerant life-history strategy commonly associated with massive coral taxa (Darling et al., 2012).

Not all early life stages of scleractinian corals exhibit high tolerance to thermal stress, and responses vary considerably among species and developmental stages. In the embryonic stage, Voolstra et al. (2009) reported that *Montastraea faveolata* embryos expressed stress-related genes under both ambient (29°C) and elevated (31.5°C) temperatures during spawning conditions in the Caribbean. Although the proportion of misshapen embryos initially increased after 12 h of exposure, it declined by 48 h, suggesting either developmental recovery or selective mortality of thermally sensitive individuals. Similarly, Keshavmurthy et al. (2014) demonstrated that extreme temperatures significantly disrupted embryonic development in *Acropora muricata* and *Acropora hyacinthus*, with low temperatures delaying fertilization and high temperatures causing deformities and elevated mortality. In contrast, the present study did not detect visible developmental impairments in embryos of *F.* *abdita* and *F. colemani* under short-term thermal exposure. This suggests that embryos of these species may be resilient to short-term warming events or localized thermal spikes. However, since the assessments were limited to morphology and developmental progression, sublethal physiological stress may have occurred despite the absence of detectable morphological abnormalities.

Phylogenetic context should also be considered when interpreting variation in thermal responses among scleractinian corals. The coral species span two major scleractinian lineages, Refertina (complex) and Vacatina (robust), which are deeply divergent evolutionary groups with distinct embryogenetic characteristics (Okubo, 2016). Comparisons across these lineages should therefore be interpreted cautiously, particularly when inferring shared mechanisms of thermal tolerance in the absence of molecular or physiological evidence. Nevertheless, the compiled studies in Table 1 indicate that phylogenetic placement alone does not consistently predict thermal responses. For example, species within Vacatina exhibited contrasting larval survival responses, with *Pocillopora damicornis* and *Lobactis scutaria* showing reduced survival under thermal stress, whereas *F. abdita* showed no apparent change. Likewise, broad functional classifications such as ‘stress tolerant’ did not consistently correspond to greater thermal tolerance, as *F. abdita* and *Favia fragum*, both classified as stress tolerant, exhibited different survival responses. Thermal tolerance also varies among coral species from different geographic locations, suggesting that species-specific and environmental factors may contribute to variation in thermal thresholds (Negri et al., 2007). These patterns suggest that thermal sensitivity reflects a combination of species-specific characteristics, phylogenetic history, and environmental context. Differences among studies in thermal exposure duration, temperature regime, and developmental stage may also contribute to the observed variation, highlighting the importance of following standardized protocols to assess thermal tolerance (Grottoli et al., 2021; Voolstra et al., 2025).

The ecological relevance of the temperature treatments summarized in Table 1 also varied among studies. Ambient or control temperatures were based on measured site conditions, spawning-season temperatures, seasonal conditions, or experimental tank conditions (Bassim et al., 2002; Randall and Szmant, 2009a; Woolsey et al., 2015; Huffmyer et al., 2024). In contrast, elevated temperatures were selected to address different experimental objectives, including temperatures within the current environmental range (Chui and Ang, 2017), projected future warming (Negri et al., 2007; Heyward and Negri, 2010; Chua et al., 2013; Olsen et al., 2013; Baria et al., 2015), upper seasonal temperatures or conditions associated with bleaching events (Randall and Szmant, 2009a,b), thermal thresholds (Woolsey et al., 2015; Da-Anoy et al., 2020), or experimentally induced heat stress (Weeriyanun et al., 2022; Alexander et al., 2022; Hazraty-Kari et al., 2023). Thus, their ecological relevance depends on how the treatment temperature relates to the environmental conditions at the study site and the specific experimental question.

No detectable effects of short-term warming were observed during the planktonic larval stages of *F. abdita* and *F. colemani*. This contrasts with other species that show increased larval mortality under elevated temperatures, including *A. tenuis* (Graham et al., 2017; Da-Anoy et al., 2020; Weeriyanun et al., 2022) and *L. scutaria* (Putnam et al., 2008). Nevertheless, the absence of mortality does not necessarily imply that larvae were unaffected by elevated temperature. For example, *Montipora capitata* larvae showed reduced respiration rates under warming conditions (Huffmyer et al., 2024), whereas *Porites astreoides* larvae showed increased respiration, oxidative stress, and antioxidant enzyme activity (Olsen et al., 2013). These findings suggest that thermal stress may induce physiological and metabolic alterations that are not immediately reflected in survival outcomes. Furthermore, thermal exposure during the larval stage does not necessarily confer enhanced tolerance later in development, as Hazraty-Kari et al. (2023) found no improvement in thermal tolerance following larval conditioning in *A. tenuis*. Taken together, these observations indicate that assessments based solely on survival may underestimate the effects of warming, highlighting the need for physiological and molecular investigations of early life stages.

The recruit stage of *Favites* spp. also tolerated short-term warming in the present study, as no significant reductions in survival were detected and no morphological abnormalities were observed in either species (representative recruits shown in Fig. 4). These findings are consistent with those reported for *Acropora kenti*, *Acropora loripes*, and their hybrids, where elevated temperatures did not significantly affect survivorship, size, coloration, or photochemical efficiency even after 5 weeks of exposure (Lamb et al., 2024). However, thermal sensitivity during recruitment has been documented in other species, including *Acropora solitaryensis* (Nozawa and Harrison, 2005) and *P. acuta* (Huffmyer et al., 2021), in which elevated temperatures resulted in significant mortality. Taken together, these results suggest that thermal tolerance during recruitment may be highly species specific. Long-term experiments incorporating growth, physiological performance, and post-settlement survival are therefore recommended to determine whether the apparent resilience of *Favites* recruits persists under prolonged or repeated thermal stress.

**Fig. 4.**
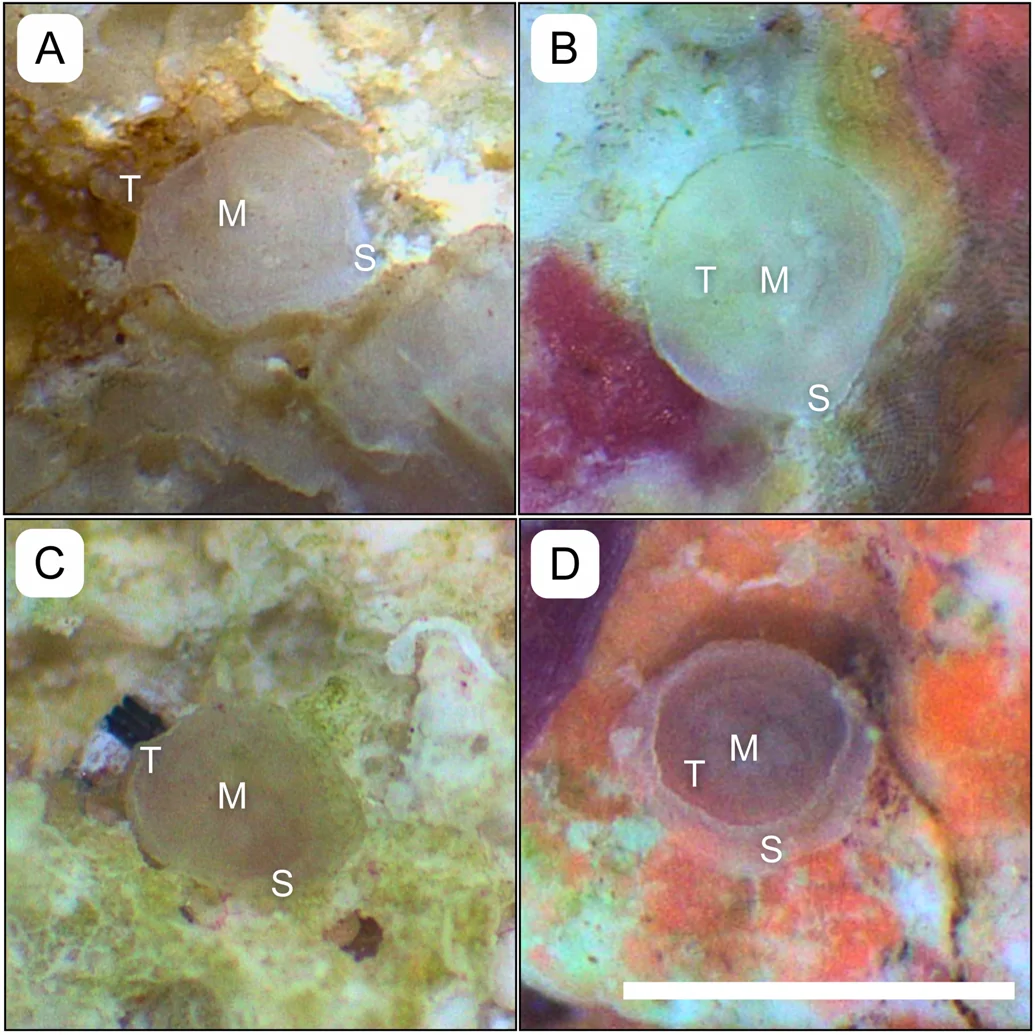
**Representative coral recruits of *F. abdita* (A,B) and *F. colemani* (C,D) 3 days after exposure to final target temperatures: control (28°C; A,C) and elevated temperature (35°C; B,D).** T, tentacles; M, mouth; S, skeleton. Scale bar: 1 mm. We applied minor global image adjustments to improve visibility.

Acute thermal stress did not significantly alter settlement dynamics in *Favites* species. No significant increase in settlement was observed at elevated temperatures. However, a small number of *F. colemani* larvae (two of 72 larvae, ≈3%) settled on the walls of culture plates at 32°C before the addition of crustose coralline algae, suggesting that these larvae may have attained settlement competency prior to the intended settlement induction period. The low frequency of these observations may reflect inadvertent transfer of water from the donor colony bins during gamete collection, despite sperm-washing procedures. Da-Anoy et al. (2016) showed that conspecific extracts can induce settlement in *P.* *damicornis* larvae, suggesting that residual water from the donor colony environment may contain chemical cues that trigger settlement responses. Alternatively, this may indicate that elevated temperature accelerated the onset of settlement competency. Similar increases in settlement under elevated temperature have been reported in several reef-building corals, including *Stylophora pistillata* (Putnam et al., 2008), *M. capitata* (Alexander et al., 2022), *Favites chinensis*, and *A. solitaryensis* (Nozawa and Harrison, 2005). This increased settlement under warming conditions could reflect earlier responsiveness to settlement cues or accelerated larval development (Nozawa and Harrison, 2000; Heyward and Negri, 2010; Raventos et al., 2021). If elevated temperature accelerates larval competency, pelagic larval duration may be reduced, potentially limiting dispersal distances and connectivity among reef habitats (Figueiredo et al., 2014). However, increased settlement does not necessarily translate into improved recruitment success. For example, *A. solitaryensis* exhibited enhanced settlement at elevated temperature but also experienced higher post-settlement mortality (Nozawa and Harrison, 2007). Settlement patterns must therefore be interpreted cautiously and within the broader context of subsequent survival bottlenecks. Increased settlement alone may not enhance overall recruitment success if post-settlement survival remains low.

In broadcast-spawning corals, the larvae are typically aposymbiotic and acquire Symbiodiniaceae following settlement during the early spat stage. Stress-tolerant algal symbionts have been shown to enhance coral thermal tolerance and improve host performance under elevated temperature conditions (Abrego et al., 2008). However, Yakovleva et al. (2009) reported that in *Acropora intermedia*, heat-stressed symbiotic larvae experienced symbiont-derived oxidative stress that caused tissue damage in the host. In the present study, *F. abdita* and *F. colemani* tolerated short-term exposure to elevated temperature (35°C) during the recruit stage, when symbiont function is not yet established. This suggests that early recruits may depend less on photosynthetic symbionts during acute thermal exposure. In contrast, thermal tolerance in adult *F. colemani* (up to ∼32°C) is closely linked to the stability of the coral-algal symbiosis and maintenance of symbiont photophysiological performance, which may increase susceptibility when symbiont function is impaired (Tañedo et al., 2021). Elevated temperature can disrupt this symbiosis by impairing symbiont photosynthesis and increasing reactive oxygen species production, ultimately contributing to bleaching and host physiological stress in symbiotic corals (Lesser, 1997; Doering et al., 2023).

Stress-tolerant corals with offspring exhibiting thermal tolerance could contribute to the potential recovery of degraded reef systems, as increased early-life thermal tolerance could improve recruitment success under warming conditions. Overall, *F. abdita* and *F. colemani* tolerated the elevated-temperature regimes tested (up to 35°C), with no consistent negative effects observed across embryonic survival and development, larval survival and settlement, and early recruit survival. However, this study examined only short-term thermal responses. We did not assess potential long-term consequences, including sublethal physiological stress, reduced skeletal growth (Cantin et al., 2010), altered symbiont acquisition (Abrego et al., 2012), and delayed mortality (Alexander et al., 2022). Recruits that appear resistant immediately after thermal exposure may still experience latent effects that influence survival, growth or competitive ability weeks to months later.

Furthermore, due to the logistical challenges of working with these specific coral life stages, we acknowledge that our sample size was limited to *n*=3 replicates per treatment group. While this limited replication reduces statistical power, evaluating nonparametric effect sizes (*r*) alongside *P*-values can provide additional context for interpreting the observed data. Following the benchmarks of Peres (2026), traits such as larval settlement in *F. abdita* and *F. colemani* fell within a large effect threshold (|*r*|≥0.5). While these descriptive metrics suggest noticeable differences between treatments, the low number of replicates means that these trends should be interpreted with caution and are intended to serve primarily as a preliminary roadmap for future replication studies. Additionally, since this study did not measure underlying metabolic or cellular responses, it remains unclear whether these statistical null results stem from a lack of thermal sensitivity or physiological changes (acclimation). Future work utilizing physiological or molecular diagnostics is required to fully resolve these underlying mechanisms.

These findings provide initial support for using early-life thermal tolerance as a screening tool within the BARC context, particularly for identifying species capable of tolerating short-term temperature stress at high temperatures (35°C). However, because these experiments were conducted under controlled laboratory conditions, they do not fully reflect the natural dynamics of reef environments. Translating this apparent tolerance into meaningful restoration outcomes requires validation under natural field conditions, where multiple environmental stressors and ecological processes such as competition, predation, resource limitation, and localized anthropogenic influences associated with ongoing mariculture activities can influence growth and survival. Future work under the BARC framework should therefore prioritize long-term and field-based monitoring of post-settlement performance, including growth trajectories, survivorship, and symbiont dynamics across varying thermal regimes. Coupling laboratory assays with *in situ* validation will provide a more comprehensive understanding of early-life resilience and improve species selection for reef rehabilitation to maximize recruitment success and persistence under ongoing climate stress.

## MATERIALS AND METHODS

### Collection and spawning monitoring of donor colonies

Fecund *Favites* spp. donor colonies were collected from the Lucero (16.411709° N, 119.904536° E) and Caniogan (16.29217° N, 120.013333° E) reefs of BARC (Fig. 5). Only healthy colonies with no signs of bleaching or disease were selected and transported to the outdoor hatchery facility of BML MSI-UPD on 10 May 2025, 2 days before the full moon. This timing was based on previously documented spawning times for these species (Maboloc et al., 2015). Prior to colony collection, we conducted rapid sampling during the last week of April 2025 to confirm their reproductive status. Colonies with white to cream-colored oocytes beneath the oral disks of polyps were considered gravid and selected for collection (Maboloc et al., 2015). Collection was permitted under the Bureau of Fisheries and Aquatic Resources Gratuitous Permit No. 0299-24, issued to the Department of Science and Technology – Philippine Council for Agriculture, Aquatic and Natural Resources Research and Development Marine Heatwaves Project.

**Fig. 5.**
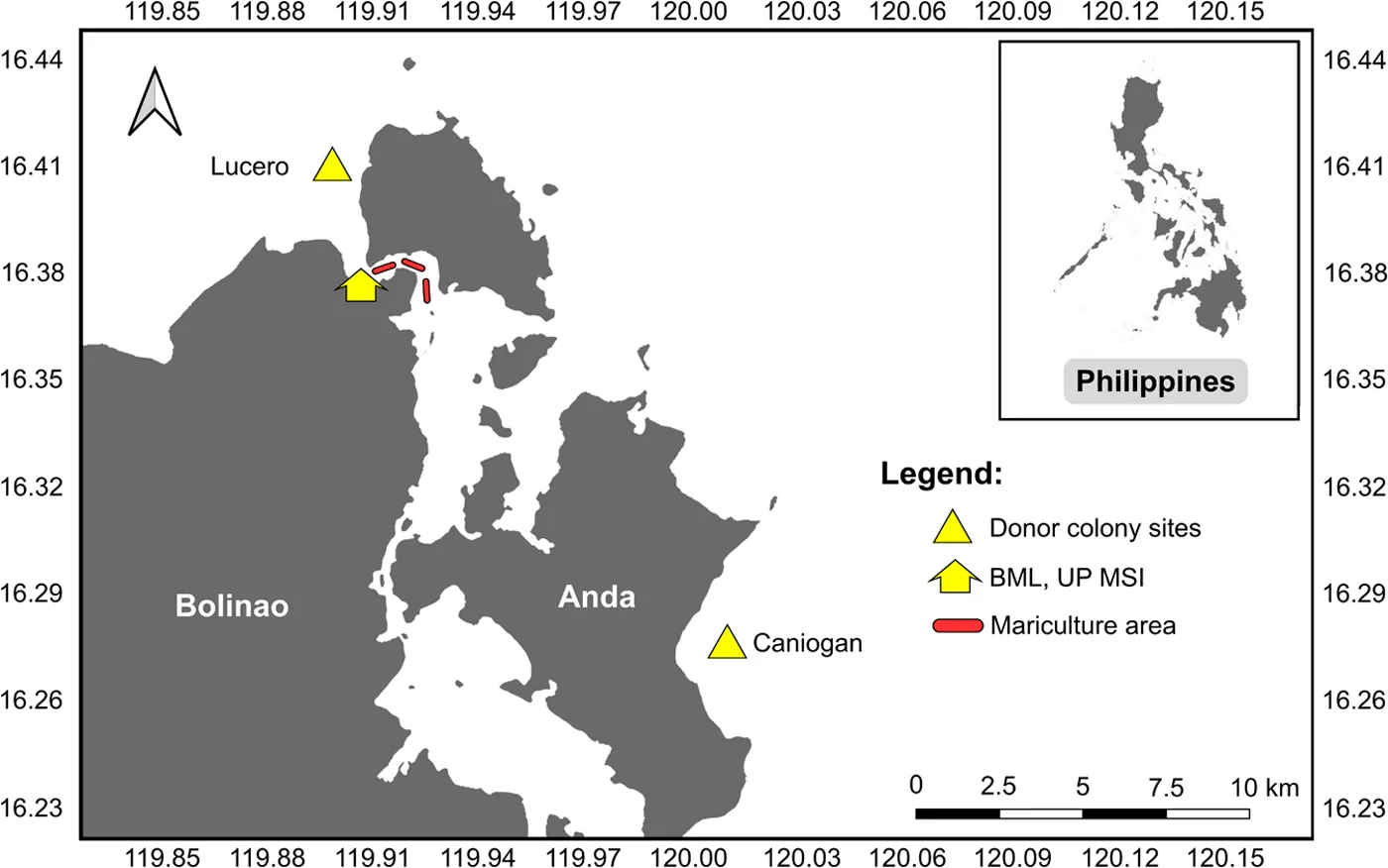
**Map of the BARC, Pangasinan, northwestern Philippines, showing the collection sites of the donor colonies indicated by the yellow triangle.** Map produced using QGIS version 3.43.3.

Donor colonies were monitored from the night of collection on 10 May 2025 until the spawning event on 16 May 2025. During this period, the colonies were transferred to individual polypropylene containers filled with UV-filtered seawater at 17:45 h and monitored for spawning from 18:00 to 23:00 h, following Maboloc et al. (2015). On 16 May 2025, 4 days after the full moon, synchronous spawning occurred. Although we initially collected a larger pool of colonies from both sites, the final experimental pool was limited to 16 colonies (eight for *F. abdita* and eight for *F. colemani*) that spawned synchronously that night and produced enough gametes to support the experiments. The colonies had geometric mean diameters of 11.08±3.17 cm for *F. abdita* and 12.29±4.57 cm for *F. colemani* (mean±s.d.) (details are summarized in Table S1). After gamete collection, we returned the donor colonies to their collection sites 5 days later.

### Fertilization and embryo-to-recruit rearing

We collected gamete bundles from individual containers using 250 ml plastic cups and bulk pooled them by transferring them into pre-labeled, clean polypropylene fertilization tanks (Nally bins) containing UV-filtered seawater. We used separate fertilization tanks for each species, with volumes of 8 l for *F. abdita* and 55 l for *F. colemani*. We combined all spawned egg-sperm bundles into these collective tanks. The bundles were gently agitated by hand to break them apart and release the sperm and eggs. After an hour, we removed excess sperm by repeatedly exchanging the seawater until it became clear, thereby minimizing the risk of polyspermy.

After fertilization, the embryos were transferred to three culture tanks (two tanks of 55 l for *F. abdita* and one tank of 125 l for *F. colemani*) for continued development. The cultures were regularly monitored and maintained at an ambient seawater temperature of 28°C under natural light conditions, following a 12:12 light-dark cycle. Water quality was maintained by performing daily 50% water changes with UV-filtered seawater until the end of the experiment. Only healthy individuals (i.e. embryos, larvae or recruits showing no deformities, abnormalities, or signs of cell disintegration) were used in the experiments. Minimal aeration was provided using aquarium air pumps connected to silicone tubing and T-control valves to prevent aggregation and adhesion to the tank walls.

### Thermal stress experimental design

Thermal stress experiments followed a single-factor (temperature), two-level (28 and 33 or 35°C) design using laboratory incubators (Maximus Max-MC001B Mini Cooler/Warmer, Philippines). We replicated each temperature treatment across three independent incubators (*n*=3). Within each incubator, two 24-well plates per species were used as subsamples, and measurements from the two plates were averaged to obtain a single incubator-level replicate. Each well contained one biological unit (embryo, larva, or recruit) in 2.5 ml UV-filtered seawater, which was randomly assigned to a treatment group (Fig. S1). Sample sizes per species and temperature treatment were 144 (embryos), 108 (larvae), and 60 (recruits). Temperature treatments were 28°C (ambient), 33°C (moderate thermal stress; +2°C above the seasonal average), and 35°C (high thermal stress; +4°C above the seasonal average). Treatment temperatures were based on *in situ* spawning season SST measurements from HOBO pendant loggers (Onset) and previous coral thermal studies (Fig. 6, Jokiel and Coles, 1990; Cziesielski et al., 2019; Conaco and Cabaitan, 2020) because the thermal tolerance of *Favites* spp. remains unknown. To contextualize these treatments, *in situ* daily average temperature records from a HOBO pendant logger (Onset) deployed at Caniogan Reef (April-May 2025; Baran, Marine Science Institute, University of the Philippines Diliman) indicated a field range of 29.5-32.2°C. The 33°C and 35°C laboratory treatments thus exceeded this observed daily average field range to simulate thermal stress on *Favites* early life stages. We verified temperature accuracy during ramping using a handheld digital thermometer (model IHT-1X, INKBIRD Tech. C.L., China). The experiment followed a short-term heat-stress protocol (Voolstra et al., 2025) and a standardized framework for assessing coral thermal responses (Grottoli et al., 2021). The temperature ramping protocols used for each early life stage are illustrated in Fig. 4A,B. A summary of the experimental design is presented in Table 2.

**Fig. 6.**
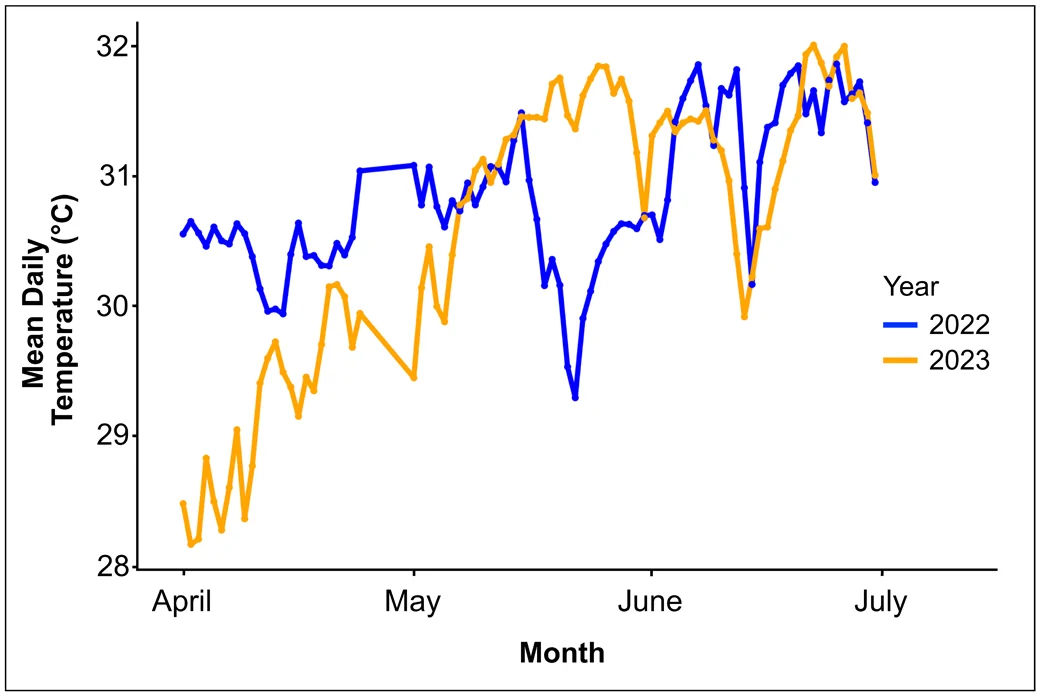
**Mean daily average temperature trend during the spawning season in 2022 and 2023 at BARC.** The combined average temperature for both years was 31.26°C (range: 27.27-33.85°C). In 2022, the temperature averaged 30.83°C (range: 28.36-33.85°C), and in 2023, it averaged 31.31°C (range: 27.27-33.54°C). Temperature data were obtained from HOBO loggers deployed by the Marine Heatwave Project of the Marine Science Institute at donor colony collection and nearby reef sites (Lucero, Caniogan, Cabunggan, Trenchera, Panacalan, RDG, and Magsaysay) at ∼5-6 m depth.

**Table 2.** Summary of the experimental design used to examine responses of early life stages of *F. abdita* and *F. colemani* to elevated temperatures

Species	Early life stage	Temperature levels (°C)	Total number of incubators	Total number of 24-well plates	Total number of biological units	Objectives	Measured parameters
*Favites abdita*	Embryos	28 (control) and 33	6	12	288	1a. Embryonic development	1a. Number of embryos developed into larvae
						1b. Embryonic survival	1b. Number of surviving embryo/larvae
	Larvae				72	2a. Larval survival	2a. Number of surviving larvae
					144	2b. Larval settlement	2b. Number of settled and metamorphosed larvae
	Recruits	28 (control) and 35			120	3. Recruit survival	3. Number of surviving recruits
*Favites colemani*	Embryos	28 (control) and 33	6	12	288	1a. Embryonic development	1a. Number of embryos developed into larvae
						1b. Embryonic survival	1b. Number of surviving embryo/larvae
	Larvae				72	2a. Larval survival	2a. Number of surviving larvae
					144	2b. Larval settlement	2b. Number of settled and metamorphosed larvae
	Recruits	28 (control) and 35			120	3. Recruit survival	3. Number of surviving recruits

Experiments were conducted using a circular LED light. During the light period (05:00–17:00 h), photosynthetically active radiation was maintained at 7.31±1.38 μmol photons m^−2^ s^−1^ as measured prior to the experiment using a light data logger (model LI-I400, LI-COR, Inc.). This low irradiance provided a controlled experimental light environment and was not intended to replicate natural daylight conditions. Dissolved oxygen (DO) and salinity were monitored using a portable DO meter (YAGO, China) and an Automatic Temperature Compensation (ATC) handheld refractometer (T3 Fish Feeds Trading, Philippines), respectively. Recorded values for temperature, DO, and salinity are provided in Table S2. Developmental observations were performed using a stereomicroscope (model SMZ-171, Motic).

### Embryo survival and development thermal experiment

Embryos (4- to 16-cell stage; ∼5 h post-fertilization) were individually transferred into 24-well plates (144 total embryos per species; Fig. S1) and placed into designated incubators. The temperature was gradually increased with an average ramping duration of approximately 5.1 h (range: 4.7-5.5 h; see Table S2) to minimize thermal shock (McLachlan et al., 2020; Voolstra et al., 2025). This ramping schedule was designed to maximize exposure at the target temperature (∼24 h) before the embryos transitioned to the actively swimming planula stage (Maboloc et al., 2015). A 12:12 light-dark cycle was maintained throughout the experiment.

Survival and developmental progression were assessed prior to each temperature ramp. Embryos that developed into actively swimming larvae were considered successfully developed. After each monitoring period, 50% of the seawater was replaced with UV-filtered seawater that had been pre-equilibrated in the incubator to match the experimental temperatures, thereby maintaining water quality and temperature stability.

### Larval survival and settlement thermal experiment

Larvae at 5 days post-fertilization were individually transferred to 24-well plates and placed in incubators. For each species, survival and settlement were assessed using separate sets of larvae. Survival assays consisted of six larvae (one per well) maintained in seawater only. The settlement assays consisted of 12 larvae (one per well) with ∼1 cm^3^ rubble containing crustose coralline algae (rCCA) and six wells for control assays: three wells with rCCA only and three wells with seawater only. The wells were randomly assigned to the respective treatments prior to adding the larvae to minimize positional effects (Fig. S1). The rCCA were collected from the field and added 4 days post-fertilization, when settlement onset was observed, following Harrington et al. (2004). The temperature in the elevated treatment (33°C) was increased stepwise (1°C every ∼5.6 h; range: 5.2-5.9 h; Table S2), maximizing exposure at the target temperature (∼24 h) during the ∼2-day experiment. Settlement was defined as successful attachment and metamorphosis, regardless of substrate. Water changes (50%) were conducted after each observation period. A 12:12 light-dark cycle was maintained throughout the experiment.

### Recruit survival experiment

On 4 days post-fertilization, 12 plates with 24 wells each were pre-loaded with ∼1 cm^3^ rCCA and UV-filtered seawater, and one larva was introduced in each. One-day-old recruits were used in the thermal stress experiment. For each species, 12 plates were prepared, each containing 10 wells with healthy spat (60 total recruits per species), six control wells (three with rCCA only and three with UV-filtered seawater only), and eight empty wells (Fig. S1). The plates were transferred to designated incubators and exposed to an elevated temperature (35°C) for ∼24 h, with an average ramping duration of approximately 23.3 h (range: 20.6-25.2 h; Table S2). This slow ramping followed the recommended rate for thermal stress studies (approximately 1°C per day; Voolstra et al., 2020; Grottoli et al., 2021). A 12:12 light-dark cycle was maintained throughout. Following each monitoring period, 50% of the seawater was replaced.

### Statistical analysis

The experiments followed a single-factor (temperature), two-level design. Because the small sample size (*n*=3 replicates per treatment group) prevents reliable testing of parametric assumptions, we used nonparametric Wilcoxon rank-sum tests to compare control (28°C) and elevated-temperature treatments (33 or 35°C). The analyses focused on embryonic and recruit survival and larval settlement. Embryo-to-larva development and larval survival were excluded from statistical analyses, as all embryos developed into larvae and no mortality or temperature effects were observed in either species. Additionally, a nonparametric effect size analysis was conducted following Peres (2026) to assess the magnitude of treatment impacts independently of sample-size-constrained *P*-values. We calculated the nonparametric rank correlation coefficient *r* for each comparison based on the normal outputs of the Wilcoxon rank-sum tests, using *n*=3 independent replicates per treatment. All analyses and visualizations were conducted in R (version 4.3.0; R Core Team, 2023, https://posit.co/) using the *tidyverse* (version 2.0.0; Wickham et al., 2019) and *patchwork* (version 1.3.1) packages.

### AI disclosure

An AI tool (Gemini) was used exclusively to assist with the generation of R code for processing and analyzing the data.

## Supplementary Material



10.1242/biolopen.062748_sup1 Supplementary information
