# Supplementary material for: Early life stages of stress-tolerant corals (Favites spp.) exhibit tolerance to acute thermal stress
Source: Biol Open. 2026 Oct 20;15(10):bio062748. doi: 10.1242/bio.062748 (PMC13730137; doi:10.1242/bio.062748)
Supplement: Supplementary file 1 [file biolopen-15-062748-s1.pdf]

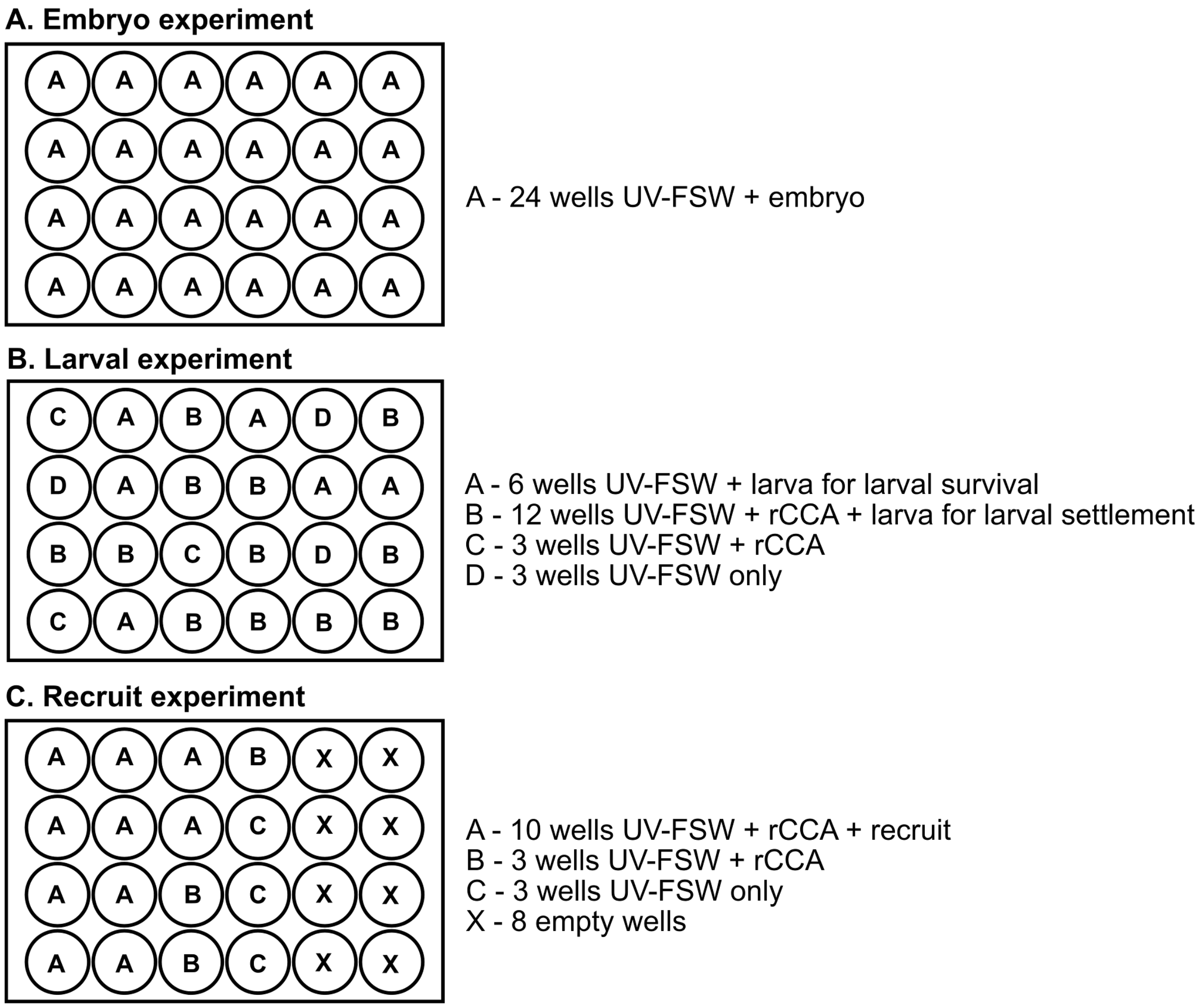

Fig. S1. Schematic representation of the 24-well plate layouts used across the three independent experiments for (A) embryos, (B) larvae, and (C) recruits. UV-FSW – UV-filtered seawater and rCCA – rubble containing crustose coralline algae.

Biology Open • Supplementary information

Table S1. Summary of donor colonies with collection site, spawning details, and morphometric measurements. Spawning categories were visually assessed based on total bundle release, independent of colony size, during the peak release window. Major spawning represents an abundant release characterized by a dense, continuous accumulation of floating gamete bundles across the water surface; Partial spawning represents an intermediate release characterized by a visibly reduced and patchy distribution of bundles; and Dribble spawning represents a minimal, scattered release characterized by a sparse distribution of single, isolated bundles.

Colony number	Species	Colony tag	Collection site	Spawning date	Spawning output	Latitude	Longitude	GD (cm)	LD (cm)	GMD	Area (cm²)
1	*Favites abdita*	001	Caniogan	16-May-25	dribble	16.29217° N	120.013333° E	7.83	4.07	5.64	25.60
2		010	Lucero	16-May-25	partial	16.411709° N	119.904536° E	14.42	9.06	11.43	1001.01
3		014	Lucero	16-May-25	dribble	16.411709° N	119.904536° E	17.28	12.58	14.75	1671.30
4		015	Lucero	16-May-25	dribble	16.411709° N	119.904536° E	16.97	13.13	14.93	1205.09
5		016	Lucero	16-May-25	dribble	16.411709° N	119.904536° E	9.31	7.32	8.25	578.70
6		020	Lucero	16-May-25	dribble	16.411709° N	119.904536° E	16.10	9.90	12.62	853.27
7		019	Lucero	16-May-25	dribble	16.411709° N	119.904536° E	16.06	8.06	11.38	878.14
8		013	Lucero	16-May-25	dribble	16.411709° N	119.904536° E	10.30	9.07	9.66	676.33
1	*Favites colemani*	001	Lucero	16-May-25	dribble	16.411709° N	119.904536° E	21.47	12.17	16.16	1583.83
2		002	Lucero	16-May-25	major	16.411709° N	119.904536° E	17.34	13.07	15.06	1609.44
3		003	Lucero	16-May-25	dribble	16.411709° N	119.904536° E	9.57	5.92	7.52	479.24
4		004	Lucero	16-May-25	dribble	16.411709° N	119.904536° E	9.54	7.80	8.63	476.41
5		005	Lucero	16-May-25	dribble	16.411709° N	119.904536° E	11.95	9.61	10.72	822.59
6		006	Lucero	16-May-25	dribble	16.411709° N	119.904536° E	8.98	6.78	7.80	482.92
7		007	Lucero	16-May-25	dribble	16.411709° N	119.904536° E	13.29	11.06	12.12	921.08
8		008	Lucero	16-May-25	dribble	16.411709° N	119.904536° E	24.43	16.93	20.34	2535.58

All spawning observations occurred within 20:00-22:00 h on May 16, 2026 (4 d after full moon). GD – Greatest diameter, LD – Least diameter, and GMD – Geometric mean diameter

Table S2. Summary of physicochemical conditions (mean ± SD) recorded during embryo, larval, and recruit thermal stress experiments under control and increased temperature treatments across monitoring intervals.

Monitoring date	Monitoring time	Temperature treatment	Exposure duration (hh:mm)	Actual temperature (°C)	DO (mg L^{-1})	Salinity (ppt)
**Embryo experiment (Start time: 5-17-2025 at 03:44)**						
5-17-2025	09:15	control (28°C)	05:31	28.07 ± 0.12	5.53 ± 0.15	34.00
	14:16	control (28°C)	05:01	28.27 ± 0.06	5.30 ± 0.26	34.00
	18:59	control (28°C)	04:43	28.20 ± 0.10	5.23 ± 0.21	34.00
5-18-2025	00:12	control (28°C)	05:13	28.00 ± 0.00	5.37 ± 0.06	34.00
	23:45	control (28°C)	23:33	28.73 ± 0.12	5.70 ± 0.10	34.00
5-17-2025	09:15	increased (28°C → 29°C)	05:31	29.60 ±0.10	5.67 ± 0.06	34.00
	14:16	increased (29°C → 30°C)	05:01	30.47 ± 0.25	5.60 ± 0.17	34.00
	18:59	increased (30°C → 31°C)	04:43	31.13 ± 0.12	4.93 ± 0.74	34.00
5-18-2025	00:12	increased (31°C → 32°C)	05:13	32.23 ± 0.15	5.57 ± 0.21	34.00
	23:45	increased (32°C → 33°C)	23:33	33.30 ± 0.20	5.17 ± 0.06	34.00
**Larval experiment (Start time: 5-19-2025 at 01:53)**						
5-19-2025	07:34	control (28°C)	05:41	28.70 ± 0.10	5.00 ± 0.30	34.00
	13:30	control (28°C)	05:56	28.23 ± 0.12	5.07 ± 0.32	34.00
	19:13	control (28°C)	05:43	28.37 ± 0.21	5.07 ± 0.32	34.00
5-20-2025	00:23	control (28°C)	05:10	28.67 ± 0.06	4.80 ± 0.70	34.00
	00:21	control (28°C)	23:58	28.97 ± 0.32	5.57 ± 0.06	34.00
5-19-2025	07:34	increased (28°C → 29°C)	05:41	29.57 ± 0.21	5.40 ± 0.17	34.00
	13:30	increased (29°C → 30°C)	05:56	30.23 ± 0.06	5.57 ± 0.67	34.00
	19:13	increased (30°C → 31°C)	05:43	31.27 ± 0.25	5.50 ± 0.26	34.00
5-20-2025	00:23	increased (31°C → 32°C)	05:10	32.40 ± 0.20	5.30 ± 0.53	34.00
	00:21	increased (32°C → 33°C)	23:58	33.20 ± 0.20	5.83 ± 0.06	34.00
**Recruit experiment (Start time: 5-23-2025 at 02:57)**						
5-24-2025	00:26	control (28°C)	21:29	28.53 ± 0.06	5.57 ± 0.06	34.00
5-25-2025	00:43	control (28°C)	24:17	28.37 ± 0.06	5.30 ± 0.26	34.00
5-25-2025	21:20	control (28°C)	20:37	28.60 ± 0.00	4.87 ± 0.29	34.00
5-26-2025	21:40	control (28°C)	24:20	28.60 ± 0.00	5.07 ± 0.15	34.00
5-27-2025	22:53	control (28°C)	25:13	28.73 ± 0.06	5.10 ± 0.26	34.00
5-28-2025	22:32	control (28°C)	23:39	28.40 ± 0.15	4.90 ± 0.10	34.00
5-29-2025	23:23	control (28°C)	24:51	28.07 ± 0.06	5.77 ± 0.20	34.00
5-24-2025	00:26	increased (28°C → 29°C)	21:29	29.27 ± 0.15	5.83 ± 0.06	34.00
5-25-2025	00:43	increased (29°C → 30°C)	24:17	30.70 ± 0.10	5.60 ± 0.17	34.00
5-25-2025	21:20	increased (30°C → 31°C)	20:37	31.23 ± 0.06	5.43 ± 0.06	34.00
5-26-2025	21:40	increased (31°C → 32°C)	24:20	32.37 ± 0.15	5.60 ± 0.10	34.00
5-27-2025	22:53	increased (32°C → 33°C)	25:13	33.30 ± 0.20	5.40 ± 0.10	34.00
5-28-2025	22:32	increased (33°C → 34°C)	23:39	34.13 ± 0.15	4.80 ± 0.10	34.00
5-29-2025	22:23	increased (34°C → 35°C)	23:51	35.23 ± 0.15	5.60 ± 0.20	34.00

Biology Open • Supplementary information

Table S3. Summary of mean (± SD) survival early life history stages of *Favites abdita* and *Favites colemani* under control (28 °C) and elevated temperature treatments (33 °C for embryos and larvae; 35 °C for recruits).

Species	Life history stage	Temperature treatment	Temperature (°C)	Mean Survival
Favites abdita	Embryo	control	28	95.83 ± 2.08
	Embryo	increased	33	92.36 ± 5.24
	Larval	control	28	100.00 ± 0.00
	Larval	increased	33	100.00 ± 0.00
	Recruit	control	28	70.00 ± 13.23
	Recruit	increased	35	78.33 ± 12.58
Favites colemani	Embryo	control	28	95.14 ± 2.41
	Embryo	increased	33	95.83 ± 2.08
	Larval	control	28	100.00 ± 0.00
	Larval	increased	33	100.00 ± 0.00
	Recruit	control	28	83.33 ± 7.64
	Recruit	increased	35	73.33 ± 2.89

Table S4. Summary of mean (± SD) settlement of the larval stage of *Favites abdita* and *Favites colemani* under control (28 °C) and elevated temperature treatments (33 °C).

Species	Temperature treatment	Temperature (°C)	Mean Settlement
Favites abdita	control	28	25.00 ± 15.02
	increased	33	58.33 ± 8.33
Favites colemani	control	28	25.00 ± 7.22
	increased	33	41.67 ± 8.33

Biology Open • Supplementary information

Table S5. Non-parametric effect size analysis of the thermal stress experiments. Effect sizes (*r*) were calculated from the normal outputs of the Wilcoxon rank-sum tests, with *n*=3 replicates per treatment group. Magnitude thresholds follow Peres (2025), where $|r| \geq 0.1$ is small, $|r| \geq 0.3$ is medium, and $|r| \geq 0.5$ is large. Dashes (-) indicate traits where statistical tests and effect sizes were omitted due to identical 100% survival and zero variance across all replicates.

Species	Early life history traits	Control temperature treatment Survival (%)	Increased temperature treatment Survival (%)	Observed difference (percentage points)	Effect size *r*	*p*-values
Favites abdita	Embryo survival	95.8	92.4	3.5	0.362	0.507
Favites colemani	Embryo survival	95.1	95.8	0.7	0.192	0.814
Favites abdita	Larval settlement	25	58.3	33.3	0.802	0.1
Favites colemani	Larval settlement	25	41.7	16.7	0.734	0.116
Favites abdita	Larval survival	100	100	0	-	-
Favites colemani	Larval survival	100	100	0	-	-
Favites abdita	Recruit survival	70	78.3	8.3	0.362	0.507
Favites colemani	Recruit survival	83.3	73.3	10	0.663	0.164

Biology Open • Supplementary information
