# Supplementary material for: Early life stages of stress-tolerant corals (Favites spp.) exhibit tolerance to acute thermal stress
Source: Biol Open. 2026 Oct 20;15(10):bio062748. doi: 10.1242/bio.062748 (PMC13730137; doi:10.1242/bio.062748)
Supplement: Supplementary file 2 [file biolopen-15-062748_review_history.pdf]

Early Life Stages of Stress-Tolerant Corals (Favites spp.) Exhibit Tolerance to Acute Thermal Stress

Jacqueline Padilla-Gamiño, Maria Vanessa Baria-Rodriguez and Jeffry Jeve
10.1242/bio.062748

Editor: Nicholas Wu

Review timeline

Original submission:	11 June 2026
Editorial decision:	18 June 2026
First revision received:	11 September 2026
Accepted:	16 September 2026

Original submission

First decision letter

MS ID#: bio.062748

MS Title: Early Life Stages of Stress-Tolerant Corals (Favites spp.) Exhibit Tolerance to Acute Thermal Stress

Authors: Jeffry Jeve; Jacqueline Padilla-Gamiño; Maria Vanessa Baria-Rodriguez

I have now reached a decision on the above manuscript.

The reviewer reports are shown at the bottom of this email.

As you will see, the reviewers raised a number of substantial criticisms that prevent me from accepting the paper at this stage. Both reviewers acknowledged that the experiment was well designed and that the manuscript reads well. My initial concern before sending this paper out to reviewers was the low sample size for the study design and organism. Reviewer 1 also agreed with this and suggested a power analysis. Unfortunately, with the low sample size (biological units are not the true sample size), detecting a true effect is difficult (also mentioned by reviewer 2), and I would suggest an additional caveat about the interpretability. Much of the interpretation depends on whether the non-significant findings reflect a true effect or a statistical artifact of a small sample size.

If the authors are able to address mine and both reviewers' concerns, I would be willing to have the paper reviewed again.

At this stage, we also ask you to ensure your manuscript complies with our formatting guidelines. Provided you are able to fully address the referees' comments, we are positive about publication of your paper (we accept over 95% of revision submissions) and therefore hope you won't mind any extra work involved in reformatting your manuscript at this point.

Please upload both a 'clean' version of your Word file, along with a highlighted version clearly showing where you have made changes in the revised manuscript. Please avoid using 'Track changes' in Word files as these are lost in PDF conversion.

© 2026. Published by The Company of Biologists under the terms of the Creative Commons Attribution License (https://creativecommons.org/licenses/by/4.0/).

I should be grateful if you would also provide a point-by-point response detailing how you have dealt with the points raised by the reviewers in the 'Response to Reviewers' box. Please attend to all of the reviewers' comments. If you do not agree with any of their criticisms or suggestions please explain clearly why this is so.

Reviewer 1

Comments for the author

The authors conduct a series of experiments to determine the sensitivity of various aspects of early life history (larval development, survival and settlement) in the robust (suborder Vacatina) corals Favites abdita and F. colemani to short term exposure to elevated temperatures.

The experiments are well designed and address an important issue: how coral recruitment and larval survival might be impacted by the increasing severity and frequency of marine heatwaves. The introduction and literature review cover the main issues, and the authors include a helpful table summarising many of the published experiments dealing with larval development and survival in response to elevated temperatures.

There are, however, some issues with the manuscript as it stands:

1. The authors present a table summarising experimental studies examining the response of different hard coral early life stages to thermal stress. While this is a useful and well researched resource, it could be somewhat misleading in that it lumps together the two suborders of Scleractinia (Complex/Refertina and Robust/Vacatina), which undergo different larval development and diverged ~460 Ma. Given this deep phylogenetic difference it would be useful to indicate to which branch each family belongs and avoid direct comparisons with evolutionarily distant species. In the absence of molecular data confirming convergent developmental and stress mechanisms it is difficult to justify comparing the Merulinidae (Vacatina) with, for example, the Acroporidae (Refertina). This phylogenetic distinction should be addressed in the discussion in addition to the functional classification as "stress-tolerant" as, without detailed molecular characterisation, it is not possible to say whether the mechanism of stress tolerance is similar in such phylogenetically divergent species.

2. The table includes the actual temperature treatments for each reported experiment, which is important. It would, however, be very useful to know how the high temperatures relate to the current temperature range for that site, i.e. is that within the current variability or does it represent a potential future?

3. Figure 3 shows the mean daily temperature (per month?), but no indication of the variability other than a single min/max temperature. It is often the variability (or lack thereof) that determines the thermal sensitivity of an organism so it would be useful to know the temperature range for each month. There is also no temperature data for the period (2025) when the experiment was conducted. This is very important information that should be presented.

4. While the experiment is well designed and the inclusion of the randomisation strategy (Fig S1) are welcome, the experiments seem underpowered in their ability to detect small to medium effects. It would be very useful to conduct a power analysis to determine the effect sizes that could be reliably detected using the existing experimental design and include this information as a caveat when discussing the lack of significant changes in larval development.

5. The authors state that response variables did not meet assumptions of normality, but do not say what tests were used to determine this, nor mention how the assumptions were violated. This is useful information that might reveal something about the response. The authors should include a histogram or density plot as supplemental data in addition to the numbers shown in Tables S3 and S4.

6. Supplementary table S1 contains very important information and should be included and discussed in the main text. In particular the "spawning output" needs to be elaborated and the

© 2026. Published by The Company of Biologists under the terms of the Creative Commons Attribution License (https://creativecommons.org/licenses/by/4.0/).

difference between "major" and "partial" and a "dribble" needs further clarification, particularly as it relates to the experimental methods. Thus, for example, were there equivalent numbers of eggs/bundles from each colony added to the fertilisation tanks, or, for example, did F. colemani colony 002 (major spawning output vs. dribble for the rest) or F. abdita colony 010 (partial vs. dribble) provide a disproportionate number of bundles? This is important information that is critical to determine whether the maternal effects of a single colony might dominate the response given that the number of eggs per colony is the limiting factor for larval production.

7. The authors state that gravid colonies were gathered from two sites, Caniogan and Lucero (Fig 2). Table S2, however, shows only a single F. abdita colony from Caniogan was used with the other 7 colonies of F. abdita and all of the F. colemani coming from Lucero. The reasons for this should be mentioned in the text and the implications discussed - for example, given that the vast majority of colonies derive from the potentially mariculture-impacted site, might those colonies be more stress tolerant than less exposed sites?

8. How were the scales in Fig. 1 determined? Was there a defined physical scale placed on the reef adjacent to the colony and included in the photograph or were the photographs taken from a defined, measured distance with a fixed lens? If the scale was added post-hoc then what corrections were applied to control for distance, parallax, etc?

Reviewer 2

Comments for the author

This manuscript examines the Bolinao-Anda Reef Complex in the Philippines, one of the regions most affected by marine heatwaves, and investigates the early life stages of Favites abdita and F. colemani, embryo development, larval settlement and survival, and recruit survival, under elevated temperature. The authors find no significant effect of increased temperature on the parameters tested and conclude that these heat-tolerant coral species produce offspring capable of withstanding repeated heat-stress events.

The manuscript is generally well written, with clearly stated hypotheses and a sound analytical approach. My main concern is whether the null results genuinely reflect thermal tolerance, or whether they instead stem from the absence of other physiological measurements that might reveal more subtle stress responses. In addition, while temperature was the manipulated variable, oxygen and salinity could act as confounding factors and deserve fuller discussion throughout the manuscript. Some sections would also benefit from edits for clarity, to make the work more accessible to the broader readership interested in early coral life-history biology. I therefore recommend minor revisions, detailed below.

Introduction

L118: both species are listed as "Least Concern" on the IUCN Red List. Although it is true, need to add „with decreasing trend" and refer to

Shlesinger, T., Nuñez Lendo, C.I., Roche, R., Crabbe, J., Huang, D., Ng, L., Porter, S.N. & Bluemel, J. 2024. Favites abdita. The IUCN Red List of Threatened Species 2024: e.T133290A165814899. https://dx.doi.org/10.2305/IUCN.UK.2024-2.RLTS.T133290A165814899.en.

and

Huang, D., Nuñez Lendo, C.I., Porter, S.N., Crabbe, J., Ng, L. & Bluemel, J. 2024. Favites colemani. The IUCN Red List of Threatened Species 2024: e.T133070A165705441. https://dx.doi.org/10.2305/IUCN.UK.2024-2.RLTS.T133070A165705441.en.

Materials and Methods:

L140: Authors need to define what a "gravid coral" is and how to identify it

© 2026. Published by The Company of Biologists under the terms of the Creative Commons Attribution License (https://creativecommons.org/licenses/by/4.0/).

L141: need to add GPS points of all sites in this paragraph

L146-147: please explain how to confirm the reproductive status in gravid colonies

L194: it is not clear why using 7 umol which is particularly low in daylight exposure. What is the PAR level`?

L207: "A 12:12 light-dark cycle", which light intensity?

L245: please mention the version of R and these packages tidyverse and patchwork.

Results:

A summary of all analysis tested and their results including p-value, F-value etc should be included in a table in the supplementeray material and referred in the text.

Discussion:

284-285: this is indeed a good point. Do you have references that parental colonies being exposed to high temperatures?

L394: expand more on the limitation of this study. For example, this study is conducted in the laboratory not reflecting natural dynamics in a natural environment. Moreover, authors need to explain if and how they tested if obtained null-results derive from a simple cause-effect or a more in-depth physiological adaptation.

Reviewer's Responses to Questions

Experimental quality

Are experiments performed using appropriate methods that will answer the question (or test the hypothesis or support the observations) posed by the authors? Is the right tool used for the job?
If 'No', please indicate reasons in Comments for Author box below.
Reviewer #1:

- Yes

Reviewer #2:

- Yes

Were the data analyzed using appropriate statistical tests?
If 'No', please indicate reasons in Comments for Author box below.
Reviewer #1:

- Yes

Reviewer #2:

- Yes

Reproducibility
Were experiments performed using adequate number of biological replicates?
If 'No', please indicate reasons in Comments for Author box below.
Reviewer #1:

- Yes

Reviewer #2:

- Yes

© 2026. Published by The Company of Biologists under the terms of the Creative Commons Attribution License (https://creativecommons.org/licenses/by/4.0/).

Is there sufficient raw data to assess the rigor of the analysis?
If 'No', please indicate reasons in Comments for Author box below.
Reviewer #1:
- No

Reviewer #2:
- No

Does the methods section provide sufficient detail to permit reproducibility?
If 'No', please indicate reasons in Comments for Author box below.
Reviewer #1:
- No

Reviewer #2:
- Yes

Completeness
Are the manuscript's conclusions supported by the data?
If 'No', please indicate reasons in Comments for Author box below.
Reviewer #1:
- Yes

Reviewer #2:
- Yes

Scholarship
Do the authors cite and discuss the merits of data that would argue for and against their conclusion?
If 'No', please indicate reasons in Comments for Author box below.
Reviewer #1:
- Yes

Reviewer #2:
- Yes

Does the manuscript title & abstract accurately reflect the contents of the manuscript, without hyperbole?
If 'No', please indicate reasons in Comments for Author box below.
Reviewer #1:
- Yes

Reviewer #2:
- Yes

First revision
Author response to reviewers' comments

Reviewer	#	Comment	Re

© 2026. Published by The Company of Biologists under the terms of the Creative Commons Attribution License (https://creativecommons.org/licenses/by/4.0/).

**R1**	1	The authors present a table summarising experimental studies examining the response of different hard coral early life stages to thermal stress. While this is a useful and well researched resource, it could be somewhat misleading in that it lumps together the two suborders of Scleractinia (Complex/Refertina and Robust/Vacatina), which undergo different larval development and diverged ~460 Ma. Given this deep phylogenetic difference it would be useful to indicate to which branch each family belongs and avoid direct comparisons with evolutionarily distant species. In the absence of molecular data confirming convergent developmental and stress mechanisms it is difficult to justify comparing the Merulinidae (Vacatina) with, for example, the Acroporidae (Refertina). This phylogenetic distinction should be addressed in the discussion in addition to the functional classification as "stress- tolerant" as, without detailed molecular characterisation, it is not possible to say whether the mechanism of stress tolerance is similar in such phylogenetically divergent species.	We thank the reviewer for highlighting the importance of phylogenetic context when comparing thermal responses among scleractinian corals. We have revised the summary table (**Table 1**) to indicate the suborder Refertina or Vacatina of each family represented in the reviewed studies, thereby providing clearer phylogenetic context for the comparisons. We have added phylogenetic contexts to acknowledge that these two major scleractinian lineages are deeply divergent and that similar thermal responses across lineages should not be interpreted as evidence for shared physiological or molecular mechanisms in the absence of supporting data in the Discussion (**Lines 360-379).**<br><br>**L360-379:** *Phylogenetic context should also be considered when interpreting variation in thermal responses among scleractinian corals. The coral species span the two major scleractinian lineages, Refertina (complex) and Vacatina*
	2	The table includes the actual temperature treatments for each reported experiment, which is important. It would, however, be very useful to know how the high temperatures relate to the current temperature range for that site, i.e. is that within the current variability or does it represent a potential future?	We thank the reviewer for this helpful suggestion. We agree that providing the environmental context of the temperature treatments is important for interpreting their ecological relevance, particularly in distinguishing whether elevated temperatures fall within the current temperature variability at the study site or represent potential future warming conditions. We considered adding an additional column to **Table 1** to indicate the basis of the ambient/control and elevated temperatures. However, given the already information-dense nature of the table, an additional column would make the table visually dense and potentially more difficult to read. We therefore

© 2026. Published by The Company of Biologists under the terms of the Creative Commons Attribution License (https://creativecommons.org/licenses/by/4.0/).

	3	Figure 3 shows the mean daily temperature (per month?), but no indication of the variability other than a single min/max temperature. It is often the variability (or lack thereof) that determines the thermal sensitivity of an organism so it would be useful to know the temperature range for each month. There is also no temperature data for the period (2025) when the experiment was conducted. This is very important information that should be presented.	We agree that presenting only the mean daily temperature (per month) and overall minimum and maximum values does not adequately describe the temporal variability in temperature. We have revised **Figure 3** to show daily average fluctuations in BARC during spawning months (with average and range records per year).<br><br>Regarding the 2025 temperature data, we utilized the 2022-2023 temperature records as the baseline historical framework to inform and justify our experimental temperature treatments during the planning phase in 2024 (as detailed in **Lines 190-195**).
	4	While the experiment is well designed and the inclusion of the randomization strategy (Fig S1) are welcome, the experiments seem underpowered in their ability to detect small to medium effects. It would be very useful to conduct a power analysis to determine the effect sizes that could be reliably detected using the existing experimental design and include this information as a caveat when discussing the lack of significant changes in larval development.	We thank the reviewer for this suggestion. We have added a non-parametric effect size analysis utilizing the rank correlation coefficient (*r*) following Peres (2025), which is now available in **Table S5**. Due to the logistically challenging nature of working with specific coral life stages, our sample size was limited to n=3 replicates per treatment group.<br><br>We have added *r*-values to the
			whether these statistical null results stem from a lack of thermal sensitivity or
	5	The authors state that response variables did not meet assumptions of normality, but do not say what tests were used to determine this, nor mention how the assumptions were violated. This is useful information that might reveal something about the response. The authors should include a histogram or density plot as supplemental data in addition to the numbers shown in Tables S3 and S4.	We did not formally test for normality in this study. While our previous statement noted that data did not meet parametric assumptions, we recognize that formal normality tests are statistically underpowered and unreliable with an n=3 sample size. Instead, we chose a non-

© 2026. Published by The Company of Biologists under the terms of the Creative Commons Attribution License (https://creativecommons.org/licenses/by/4.0/).

	6	Supplementary table S1 contains very important information and should be included and discussed in the main text. In particular the "spawning output" needs to be elaborated and the difference between "major" and "partial" and a "dribble" needs further clarification, particularly as it relates to the experimental methods. Thus, for example, were there equivalent numbers of eggs/bundles from each colony added to the fertilisation tanks, or, for example, did *F. colemani* colony 002 (major spawning output vs. dribble for the rest) or *F. abdita* colony 010 (partial vs. dribble) provide a disproportionate number of bundles? This is important information that is critical to determine whether the maternal effects of a single colony might dominate the response given that the number of eggs per colony is the limiting factor for larval production	We thank the reviewer for this critical observation regarding potential maternal bias. We have chosen to retain **Table S1** in the supplementary material to maintain a concise main text, but we have expanded our text to elaborate directly on the spawning outputs and their implications. Spawning outputs (dribble, partial, and major) were also defined in the Table caption.<br><br>To be fully transparent, we did not equalize or standardize the volume of gametes from each colony; all spawned egg-sperm bundles were combined into the collective fertilization tanks.<br><br>Consequently, it is correct that more productive individuals, such as *F. colemani* colony 002 (major output) and *F. abdita* colony 010
			maternal effects are critical to understanding offspring
	7	The authors state that gravid colonies were gathered from two sites, Caniogan and Lucero (Fig 2). Table S2, however, shows only a single *F. abdita* colony from Caniogan was used with the other 7 colonies of *F. abdita* and all of the *F. colemani* coming from Lucero. The reasons for this should be mentioned in the text and the implications discussed - for example, given that the vast majority of colonies derive from the potentially mariculture-impacted site, might those colonies be more stress tolerant than less exposed sites?	We thank the reviewer for this valuable observation. A larger pool of donor colonies was originally collected from both Lucero and Caniogan. However, our final experimental pool was restricted to the 16 specific colonies that spawned synchronously on the night of 16 May 2025 and produced a sufficient volume of gametes, from the same cohort, to support the experimental treatments.<br><br>**Lines 153-163** in the methods section has been revised to clarify this selection process. Additionally, we have added a statement in the discussion in **Lines 315-323** to address the potential implications of this site distribution. Because the majority of our experimental colonies originated from Lucero, we explicitly note that their baseline thermal tolerances

© 2026. Published by The Company of Biologists under the terms of the Creative Commons Attribution License (https://creativecommons.org/licenses/by/4.0/).

	8	How were the scales in Fig. 1 determined? Was there a defined physical scale placed on the reef adjacent to the colony and included in the photograph or were the photographs taken from a defined, measured distance with a fixed lens? If the scale was added post-hoc then what corrections were applied to control for distance, parallax, etc?	We acknowledge the concern regarding scale calibration. **Fig. 1** was intended for qualitative species identification and morphological comparison of the colonies used in this study. No standardized physical scale or fixed imaging distance was used during image acquisition; therefore, reliable spatial
**R2**	1	L118: both species are listed as "Least Concern" on the IUCN Red List. Although it is true, need to add "with decreasing trend" and refer to Shlesinger, T., Nuñez Lendo, C.I., Roche, R., Crabbe, J., Huang, D., Ng, L., Porter, S.N. & Bluemel, J. 2024. *Favites abdita*. The IUCN Red List of Threatened Species 2024: e.T133290A165814899.https://dx.doi.org/10.2305/IUCN.UK.2024-2.RLTS.T133290A165814899.en. and Huang, D., Nuñez Lendo, C.I., Porter, S.N., Crabbe, J., Ng, L. & Bluemel, J. 2024. *Favites colemani*. The IUCN Red List of Threatened Species 2024: e.T133070A165705441. https://dx.doi.org/10.2305/IUCN.UK.202	We incorporated the suggestion of adding the decreasing population trend of both species and cited the references (**Lines 116-118**)<br><br>**L116-118:** *Both species are currently listed as “Least Concern” on the IUCN Red List (IUCN, 2025)*
	2	L140: Authors need to define what a "gravid coral" is and how to identify it	This was addressed in **Lines 147-149**<br><br>**L147-149:** *Colonies*
	3	L141: need to add GPS points of all sites in this paragraph	GPS coordinates were added for collection sites in **Lines 141-142:** *“Lucero (16.411709° N*
	4	L146-147: please explain how to confirm the reproductive status in gravid colonies	Inserted this statement in **Lines 147-149**: *“Colonies exhibiting white to cream oocytes beneath the oral*
	5	L194: it is not clear why using 7 umol which is particularly low in daylight exposure. What is the PAR level?	Thank you for pointing this out. We have clarified the PAR level and the low-light condition used in **Lines 205-209**. The measured PAR level was 7.31 ± 1.38 μmol photons m^{-2} s^{-1}. This low-light level provided a controlled, low-irradiance environment during the experimental period and was not
	6	L207: "A 12:12 light-dark cycle", which light intensity?	We have included the irradiance conditions of the experiment in

© 2026. Published by The Company of Biologists under the terms of the Creative Commons Attribution License (https://creativecommons.org/licenses/by/4.0/).

	7	L245: please mention the version of R and these packages tidyverse and patchwork.	We have added the versions of R and the packages used (**Lines 264-265**). We have also added more information on the non-parametric analysis conducted following Peres (2025) (**Lines 259-265**).<br><br>**L249-265:** *Additionally, a non-*
	8	A summary of all analysis tested and their results including p-value, F-value etc should be included in a table in the supplementary material and referred in the text.	We agree with the reviewer's suggestion to include a comprehensive summary table. Because our experimental design relied on non-parametric Wilcoxon
	9	L284-285 (L303-304 in the revised manuscript): this is indeed a good point. Do you have references that parental colonies being exposed to high temperatures?<br><br>**L303-304:** *While the environmental history of parent colonies may have contributed to this response, the extent of such effects remains uncertain and likely interacts….*	We have added two citations (Edullantes et al., 2023 & Francisco et al., 2025) in **Lines 310-311** to provide regional context for the thermal history of our source colonies. Edullantes et al. (2023) characterized MHWs in the Philippines and identified the West Philippine Sea, where BARC is located, as a critical MHW hotspot. Francisco et al. (2025) further detailed the spatiotemporal evolution of MHWs in the Philippines during the 2023/24 El Niño event, which coincided with the 4th global mass coral bleaching event and resulted in regional sea surface temperatures that surpassed previously recorded historical thresholds.<br><br>**L310-311:** *However, this tolerance cannot be attributed*
	10	L394: expand more on the limitation of this study. For example, this study is conducted in the laboratory not reflecting natural dynamics in a natural environment. Moreover, authors need to explain if and how they tested if obtained null-results derive from a simple cause- effect or a more in-depth physiological adaptation.	We have inserted the limitations of the study in **Lines 486-488**. We also expanded the discussion in **Lines 471-483** to emphasize the limitations and explicitly address the differences between controlled laboratory settings and complex natural environments.<br><br>**L486-488:** *However, because*

Additional Revisions by the Authors:

- In **Figure 4**, Panel C was revised to present original images separated by species with their original backgrounds. No image content was altered.

© 2026. Published by The Company of Biologists under the terms of the Creative Commons Attribution License (https://creativecommons.org/licenses/by/4.0/).

Images were acquired using an iPhone 13 Pro and an OM System Tough TG-7 digital camera through as stereomicroscope. Scale bars were removed because they were derived from a separate image and were not intended to represent the scale of the photographs shown. The figure is provided as a qualitative illustration of developmental stages.

- In **Figure 5,** we increased the font size of text labels
- In **Figure 6**, we added a statement in the figure caption to acknowledge minor global image adjustments made to improve visibility of recruits

---

Second decision letter

MS ID#: bio.062748R1

MS Title: Early Life Stages of Stress-Tolerant Corals (Favites spp.) Exhibit Tolerance to Acute Thermal Stress

Authors: Jeffry Jeve; Jacqueline Padilla-Gamiño; Maria Vanessa Baria-Rodriguez

I am happy to tell you that your manuscript has been accepted for publication in Biology Open, pending our standard publication integrity checks. It was accepted on 16th September 2026.

© 2026. Published by The Company of Biologists under the terms of the Creative Commons Attribution License (https://creativecommons.org/licenses/by/4.0/).
